# Sex differences in macro- and microvascular reactivity – a systematic review and meta-analysis

**DOI:** 10.1016/j.ajpc.2025.101335

**Published:** 2025-10-25

**Authors:** Ádám Fekete, Dániel Horváth, Patrik Kreuter, Petrana Martinekova, Eszter Ágnes Szalai, Gergely Agócs, Péter Sótonyi, Gábor Varga, Péter Hegyi, Beáta Kerémi, János Vág

**Affiliations:** a Centre for Translational Medicine, Semmelweis University, Baross u 22, 1085, Budapest, Hungary; b Department of Restorative Dentistry and Endodontics, Semmelweis University, Szentkirályi u. 47, Budapest 1088, Hungary; c Department of Oral Diagnostics, Semmelweis University, Szentkirályi u. 47, Budapest 1088, Hungary; d Department of Paediatric Dentistry and Orthodontics, Semmelweis University, Szentkirályi u. 47, Budapest 1088, Hungary; e Institute for Clinical and Experimental Medicine, Vídeňská 1958, Prague 140 21, Czechia; f Department of Biophysics and Radiation Biology, Semmelweis University, Tűzoltó u. 37-47., Budapest 1094, Hungary; g Department of Vascular and Endovascular Surgery, Semmelweis University, Városmajor u. 68, Budapest 1122, Hungary; h Department of Oral Biology, Semmelweis University, Nagyvárad tér 4, Budapest 1089, Hungary; i Institute of Pancreatic Diseases, Semmelweis University, Tömő utca 25-29, Budapest 1083, Hungary; j Institute for Translational Medicine, Medical School, University of Pécs, Pécs Szigeti utca 12, Hungary

**Keywords:** Gender, Dilatator, Vascular health, Shear stress, Flow mediated, Shear mediated

## Abstract

**Introduction:**

Sex differences in cardiovascular disease are well recognized, but underlying vascular mechanisms remain unclear. This meta-analysis investigates whether young adult healthy males and females differ in macro- and microvascular reactivity, via flow-mediated dilation (FMD) and post-occlusive reactive hyperemia (PORH).

**Methods:**

A systematic search of MEDLINE, Embase, and Central identified eligible studies meeting the criteria: FMD or PORH test assessment; Ultrasound or Laser Doppler was used on the brachial artery or the skin; including both sexes under the mean age of <40 years and a mean BMI of <30. We performed a quantitative analysis for baseline artery diameter (BAD), FMD, wall shear rate (WSR), relative change in blood flow on the brachial artery (ΔBF) and microcirculation (microΔBF), and time-to-peak (TTP). Random-effects model was used for synthesis with sex as a predictor and age and BMI as moderators.

**Results:**

A total of 51 eligible articles comprising 2426 subjects were included. Without adjustment for age and BMI (studies: 48), females had smaller BAD and TTP but higher FMD and WSR compared with males. No significant differences were found for ΔBF or microΔBF. After adjustment for age and BMI and inclusion of studies using forearm occlusion (studies: 23), FMD differences between sexes were no longer significant.

**Conclusion:**

While WSR was consistently higher in young females than in males, there was no clear evidence that females exhibit higher FMD after forearm occlusion. Likewise, greater vascular reactivity in microcirculation could not be statistically confirmed.

**Abbreviations**
ΔBF: Relative change in blood flow on the brachial artery
BAD: Baseline artery diameter
BMI: Body mass index
CI: Confidence interval
CVD: Cardiovascular disease
FMD: Flow-mediated dilation
microΔBF: Relative change in blood flow in microcirculation
PORH: Post-occlusive reactive hyperemia
WSR: Wall shear rate
WSR AUC: Wall shear rate area under the curve

**KEYS POINTS**

Flow-mediated dilation (FMD) is a widely accepted noninvasive tool for assessing endothelial function and vascular health.

This meta-analysis provides robust evidence of sex-related differences in vascular reactivity in healthy populations under the age of 40.

Females below the age of 40 show significantly greater FMD than males, but there is no clear evidence for this, after adjusting for age and BMI.

Alt-text: Unlabelled box

## 1 Introduction

Cardiovascular diseases (CVD) are the leading cause of death globally, accounting for 20.5 million deaths in 2021 and placing a major burden on health systems worldwide [1]. The reactive hyperemia test shows promise as a predictive tool for CVDs [2,3]. However, the role of sex in influencing its outcomes remains unclear [4], with studies variably suggesting higher reactivity in males [5,6], in females [7], no difference at all [8], or attributing differences to baseline vascular characteristics [9]. Younger women have a lower age-adjusted prevalence of CVD compared to men [[10], [11], [12], [13]]. Understanding how sex affects vascular reactivity is crucial for accurate assessment and prediction.

CVD mortality rates for women under 55 are increasing, in contrast to the decreasing trend observed in men [[10], [11], [12], [13]]. The difference in age-adjusted disease prevalence disappears after menopause, suggesting the influence of sex hormones. It is thought that 17β-estradiol and its receptors have a protective effect on cardiovascular physiology and pathophysiology [[14], [15], [16]]. Endothelial cells are targets for 17β-estradiol, as 17β-estradiol receptors are also present on endothelial cells [17,18]. They mediate nitric oxide and prostanoid production [19,20], increase angiogenesis and repair [17], and protect endothelial cells from shock conditions and reactive oxygen species [21,22]. As a result, the endothelium plays a crucial role in regulating macrovascular and microvascular blood flow. Consequently, endothelial dysfunction may trigger the onset of CVDs [23,24]. Additionally, endothelial dysfunction is associated with hematologic, renal, and rheumatologic diseases, erectile dysfunction, and diabetes mellitus [[25], [26], [27]].

Celermajer et al. [28] developed the Flow Mediated Dilation (FMD) technique to evaluate macrovascular endothelial function in the brachial artery by ultrasound Doppler measurement. Briefly, the release of cuff occlusion results in an increase in blood flow in the brachial artery, which increases the wall shear rate on the endothelium, leading to the release of vasodilator substances and, consequently, vessel dilation. Since then, efforts have been made to standardize the methodology across studies, supported by guidelines [29] and a recent expert consensus [30]. While flow-mediated dilation (FMD) was initially developed using Doppler ultrasound techniques, advancements have led to the development of oscillometric methods, such as enclosed-zone FMD (ezFMD) [31], which offer comparable [32] or even superior performance in certain aspects [31,32]. However, Doppler ultrasound remains the gold standard for assessing endothelial function due to its established reliability and widespread clinical acceptance [33].

The similar technique, Post Occlusive Reactive Hyperemia (PORH), can assess skin microvascular function using laser Doppler methods [34], as it is easily accessible for non-invasive measurements and has been proven to be a reliable model of disease progression [35].

Celermajer et al. [36] also demonstrated a steep decrease in FMD after the age of 41 years in men and 53 years in women. This decrease in females is progressing through the stages of menopause [37], resulting in a faster decline than in males [38]. The transition to menopause is associated with an increased risk of developing diseases, such as diabetes mellitus [39], obesity [40], chronic kidney disease [41], Alzheimer’s [42], osteoporosis [43], and accelerated vascular aging that increases the risk of CVD [44,45]. Endothelial dysfunction is present in all of these diseases, which may explain why young, premenopausal women are “protected” against such diseases and why they are underrepresented in disease trials [46].

This systematic review and meta-analysis aimed to compare baseline artery diameter (BAD), FMD, wall shear rate (WSR), relative changes in blood flow in the brachial artery (ΔBF) and skin (microΔBF), and time-to-peak artery diameter (TTP) between young, healthy adult females and males. We tested the null hypothesis that no significant sex differences exist in these measures, independent of age, and that any observed effects persist after adjusting for BMI.

## 2 Methods

This systematic review and meta-analysis was based on the PRISMA 2020 guideline [47] (see Supplementary Table 1) and followed the Cochrane Handbook [48]. The study protocol was registered with PROSPERO (CRD42023479261).

### 2.1 Eligibility criteria

The following population met our inclusion criteria: (a) including both sexes with the mean age of 40 and less, (b) generally considered healthy by the authors, c) non-smokers, and non-obese (Body mass index: (BMI)18–30). Articles were excluded if they included only postmenopausal women or elderly subjects, subjects with systemic disease without controls, subjects regularly taking any medication other than oral contraceptives, obese (BMI>30), or had a history of smoking.

The studies were considered eligible if (a) FMD or PORH test; (b) was carried out on the brachial artery or the skin; (c) the test was evaluated with Ultrasound Doppler or Laser Doppler; and (d) if the study was two-armed, including both sexes. Language was not restricted to English. Reviews, meta-analyses, commentaries, letters to the editor, and conference abstracts were excluded.

### 2.2 Information sources

Multiple electronic databases (MEDLINE, Embase, and Central) were searched for initial identification of relevant studies from inception until November 30, 2023. The search strategy can be found in Supplementary Document S1.

### 2.3 Selection process

After pilot screening and discussion, independent reviewers assessed the eligibility of all studies. Two reviewers (ÁF, DH) conducted initial title and abstract screening separately. Disagreements between the two reviewers were resolved by consultation with a third investigator (PM). The full-text selection was conducted by three reviewers separately (ÁF, DH, PK). As in the previous step, disagreements were resolved by consultation with a fourth investigator (PM). The same method was used during reference checking.

## 3 Data collection process and data items

Data from each study were extracted in pairs by the co-authors (ÁF, DH, PK) into a predefined Excel sheet.

Participant characteristics included sample size, age, hormonal status, BMI, and smoking status. The length and location of the FMD or PORH test were also collected. Extracted outcome measures included BAD, peak brachial artery diameter, absolute and relative diameter change (considered as FMD, the primary outcome), baseline and peak brachial blood flow, and ΔBF. When available, WSR and TTP values were also extracted.

For Laser Doppler Flowmetry studies on the skin, microΔBF was extracted following cuff release.

### 3.1 Study risk of bias assessment

The Joanna Briggs Institute (JBI) Critical Appraisal Checklist for Analytical Cross-sectional Studies [49] was used to evaluate the risk of bias in the identified studies, and the assessment outcomes were visually represented. The risk of bias was assessed independently by three researchers (ÁF, DH, and PK); any disagreements were resolved through consensus. The eight questions on the checklist were answered in accordance with predefined key points. See Document S2.

### 3.2 Synthesis methods

We conducted a comprehensive meta-analysis to evaluate the relationship between outcomes and potential moderators of vascular responses across several hemodynamic parameters. Macrocirculatory outcome parameters were: BAD in mm, FMD in %, ΔBF in %, WSR as area under the curve (AUC), and TTP in seconds. There was also a microcirculatory outcome parameter: microΔBF in %. All these outcomes are continuous variables. Moderators included sex (our primary moderator of interest), cuff position (forearm or upper arm), age (only studies with a mean age below 40 years were included), and BMI. Entries including smokers or patients with unknown/undeclared smoking status were excluded from the meta-analysis. Distribution of both moderators and outcomes was checked using bar plots or histograms.

All extracted data entries were screened for completeness. Each outcome variable was assessed in both long format (with the effect size measure being the mean) and wide format (male–female comparison with the effect size measure being the mean difference). For explorative purposes, overview meta-analyses and forest plots were prepared for each outcome in both formats; sex was used for subgrouping (i.e., as a categorical moderator) in the long format.

Effect sizes were pooled using random-effects models with restricted maximum likelihood estimation and Hartung–Knapp adjustment to account for between-study heterogeneity and to provide more robust confidence intervals. Between-study variance (τ²) and heterogeneity (I²) were estimated and reported for each model.

To explore potential moderators, we fitted multilevel meta-regression models using the metafor::rma.mv() function, with a random intercept for each study. Moderators to be included were selected and ranked based on their clinical significance and prior knowledge. Random effects were specified using a nested structure; for the long format, it was study/entry pair/entry; for the wide format, it was study/entry pair (entry pair representing a male–female pair). Continuous moderators generally showed a certain amount of spread, the mean values were used as representative values.

Assessment of publication bias was performed by visual inspection of funnel plots, complemented by formal statistical tests. Egger’s regression test was conducted when ≥10 studies were available, and additionally, Begg’s rank correlation test was applied to assess funnel plot asymmetry. To further evaluate the robustness of findings against potential small-study effects, we conducted trim-and-fill analysis, which we interpreted in two ways: (1) as a sensitivity analysis adjusting pooled estimates, and (2) as an estimation of the number of potentially missing studies with negative results.

Predicted values and confidence intervals from the meta-regression models were visualized using scatter plots stratified by sex and plotted against either age or BMI (non-plotted continuous predictors were fixed at the mean value across all included entries).

Analysis was performed using R (version 4.4.3) using the metafor (version 4.8.0) [50], meta (version 8.0.2) [51], and dmetar (version 0.1.0) [52] packages.

Fig. 1

**Fig. 1:**
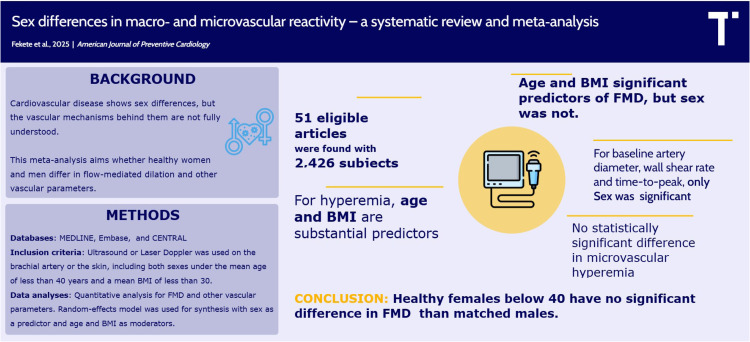
Central illustration.

## 4 Results

### 4.1 Search and selection

**Fig. 2:**
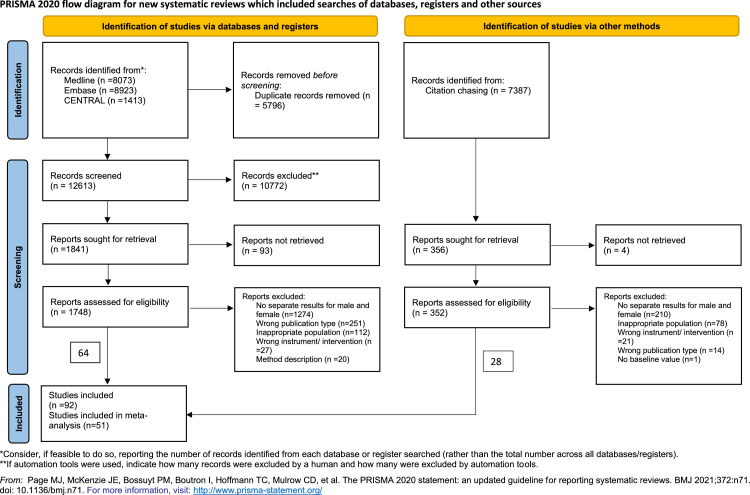
PRISMA 2020 flowchart representing the study selection process.

The initial search yielded 18,409 articles. As a result, 1841 articles remained for full-text review. During the full-text selection, 64 articles were found eligible for inclusion. At this stage, citation-chasing was conducted, and 28 more articles were found eligible. Of the 92 eligible full-text articles, we decided not to include 41 articles for the following reasons: 23 articles failed to mention smoking status, 9 articles had a wide age range (using adolescents or probably post-menopausal women and age-matched men), 7 included non-poolable outcome measures, and 2 had missing data. For the list of these articles, see Table S3. Fifty-one were included in the meta-analysis (Fig. 2).

### 4.2 Basic characteristics of included studies

Baseline characteristics of the enrolled studies are detailed in Tables S4A and S4B. The included studies were cross-sectional, prospective cohort, and crossover studies. They originated from North and South America, Europe, and Asia. All individuals in the meta-analysis were healthy non-smokers, with the mean age and BMI of the participants ranging from 19.5 to 38 years and 19 kg/m^2^ to 30 kg/m^2^. The overall mean age and BMI were 27.5 years and 23.8 kg/m^2^. (Table S4–5)

Both univariate and multivariate analyses were performed for the different outcomes. Although a larger number of studies were eligible for the univariate analyses, these models yielded less precise estimates. In contrast, the multivariate analyses provided more robust predictions and are therefore reported in detail below.

### 4.3 Baseline artery diameter (BAD)

**Fig. 3:**
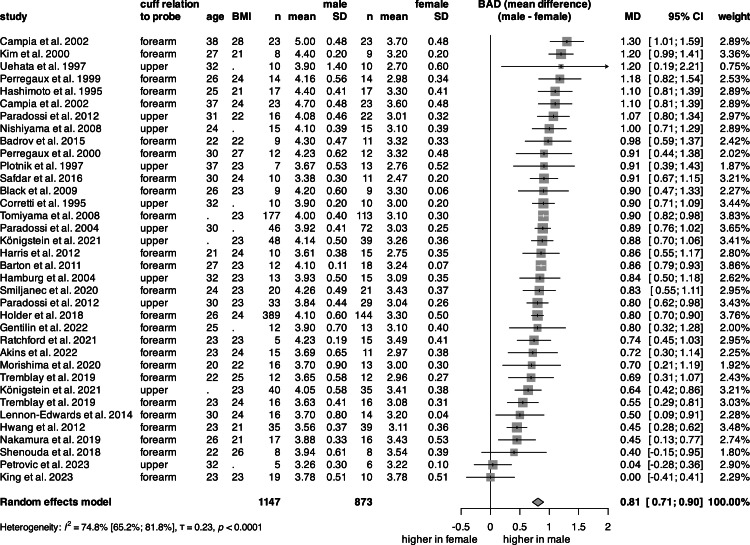
**Forest plot of the Mean difference in BAD between sexes.** Vertical lines represent the pooled point estimate, horizontal lines represent the 95 % CI, and squares represent the weight for each study. Diamond represents the pooled mean difference in BAD between sexes. (BMI: body mass index, n: count, SD: standard deviation, BAD: baseline artery diameter, 95 % CI: 95 % confidence interval).

The univariate analysis of BAD (Fig. 3, S1) was conducted on 2020 subjects from 33 articles. The mean BAD for females was 3.17 mm, and 3.99 mm for the males. The pooled mean BAD across sexes was 3.57 mm (Fig. S1). The male BAD was significantly, 0.81 mm larger than that of the female (Fig. 3).

In the multivariate linear regression analysis, 1049 subjects were analyzed (39 % female) (Table 1, Fig. S2). The regression model included sex, mean age, and mean BMI. The model indicated that male has significantly larger BAD, but age and BMI were not significant predictors of BAD.

**Table 1:** Multivariate linear regression analysis of mean FMD, mean BAD, mean ΔBF, mean WSR, and mean TTP. Significant values are highlighted in bold. (FMD: flow-mediated dilation, BAD: baseline artery diameter, ΔBF: relative change in blood flow, WSR: wall shear rate, TTP: time-to-pek, BMI*: body mass index, CI†: confidence interval).

Measured outcome	Demography		Sex		Age			BMI		
	Sex	N	β (male-female) [95 % CI]	p-value	Age (SD)	β [95 % CI]	p-value	BMI (SD)	β [95 % CI]	p-value
FMD	female	479	−0.009 [−0.019 ‒ 0.020]	0.110	25.3 (4.0)	**0.003** **[0.001–0.005]**	**0.002**	22.59 (1.74)	**−0.008** **[−0.012 ‒ −0.004]**	**<0.001**
	male	698			25.2 (3.4)			23.59 (1.38)		
BAD	female	410	**0.795** **[0.667 ‒ 0.922]**	**<0.000**	24.5 (3.6)	0.003 [−0.034 ‒ 0.028]	0.866	22.44 (1.72)	0.000 [−0.054 ‒ 0.054]	0.999
	male	639			25.1 (3.4)			23.68 (1.43)		
ΔBF	female	72	0.803 [−0.341 ‒ 1.946]	0.169	26.2 (2.9)	**−0.635** **[−1.057 ‒ 0.213]**	**0.003**	21.28 (1.63)	**−0.482** **[−0.956 ‒ 0.008]**	**0.046**
	male	75			26.4 (2.7)			23.44 (1.53)		
WSR	female	281	**−3661.539** **[−6196.760 ‒ −1126.318]**	**0.005**	23.1 (3.5)	41.750 [−1029.460 ‒ 1112.960]	0.939	23.14 (1.27)	−326.228 [−2627.239 ‒ 1974.784]	0.781
	male	517			23.9 (2.6)			23.5 (1.05)		
TTP	female	245	**6.909** **[2.632 ‒ 11.186]**	**0.002**	21.9 (2.3)	0.853 [0.166 ‒ 1.871]	0.101	23.18 (1.36)	−1.855 [−3.883 ‒ 0.173]	0.073
	male	480			22.7 (1.7)			23.82 (1.64)		

### 4.4 Flow-mediated dilation (FMD)

**Fig. 4:**
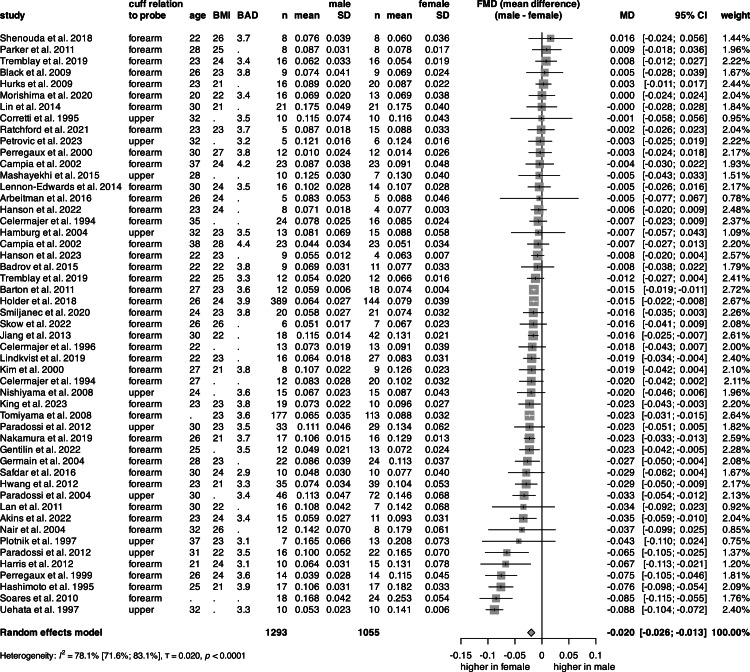
**Forest plot of the mean difference in FMD between sexes.** Vertical lines represent the pooled point estimate, horizontal lines represent the 95 % CI, and squares represent the weight for each study. Diamond represents the pooled mean difference in FMD between sexes. FMD values are expressed as relative changes. (BMI: body mass index, BAD: baseline artery diameter, n: count, SD: standard deviation, FMD: flow-mediated dilation, 95 % CI: 95 % confidence interval).

The univariate analysis of FMD (Fig. 4, S5) was conducted on 2348 subjects from 48 articles. The mean FMD was 10.4 % for females and 8.3 % for males. The pooled mean FMD of healthy individuals across sexes was 9.3 % (Fig. S5). Male participants exhibited a significantly lower FMD compared with females, corresponding to a mean absolute difference of about two percentage points. (Fig. 4).

In the multivariate linear regression, data from 1205 subjects (41 % female) were analyzed (Table 1, Fig. S6). The model, which included sex, mean age, and mean BMI, indicated that sex had no significant effect on FMD, whereas age and BMI were significant predictors

### 4.5 Relative change in blood flow on the brachial artery (ΔBF)

**Fig. 5:**
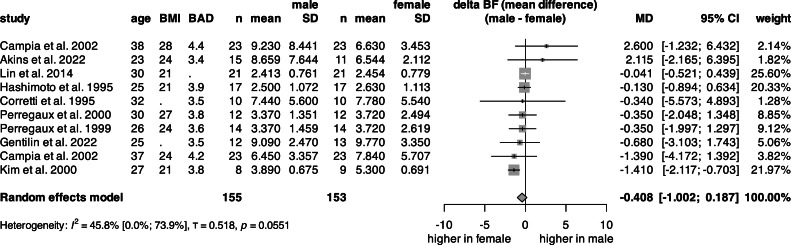
**Forest plot of the mean difference in ΔBF between sexes.** Vertical lines represent the pooled point estimate across sexes, horizontal lines represent the 95 % CI, and squares represent the weight for each study. Diamond represents the pooled mean difference in ΔBF between sexes. (BMI: body mass index, BAD: baseline artery diameter, n: count, SD: standard deviation, ΔBF: relative change in blood flow on the brachial artery, 95 % CI: 95 % confidence interval).

**Fig. 6:**
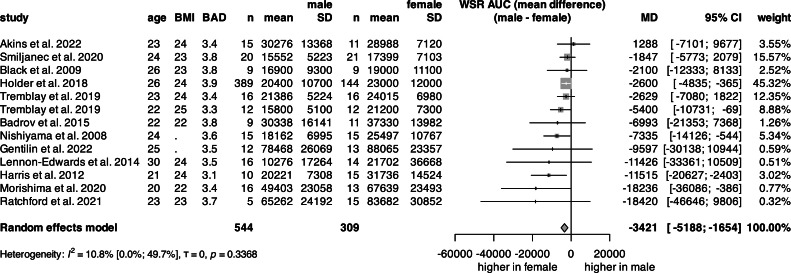
**Forest plot of the mean difference in WSR between sexes.** Vertical lines represent the point estimate, horizontal lines represent the 95 % CI, and squares represent the weight for each study. Diamond represents the pooled mean difference in WSR between sexes. (BMI: body mass index, BAD: baseline artery diameter, n: count, SD: standard deviation, WSR AUC: wall shear rate area under the curve, 95 % CI: 95 % confidence interval).

The univariate analysis of ΔBF (Fig. 5, S9) was conducted on 308 subjects from 9 articles. The mean ΔBF for females was 546.4 %, and 530.3 % for males. The pooled total value of the mean ΔBF was 536.7 % (Fig. S9). There was a non-significant 40.8 % difference, indicating a tendency toward higher hyperemia in females (Fig. 6).

In the multivariate linear regression, data from 147 subjects (49 % female) were analyzed (Table 1, Fig. S10). The model, which included sex, mean age, and mean BMI, showed that sex had no significant effect on ΔBF, while both age and BMI were significant predictors.

### 4.6 Wall shear rate (WSR)

**Fig. 7:**
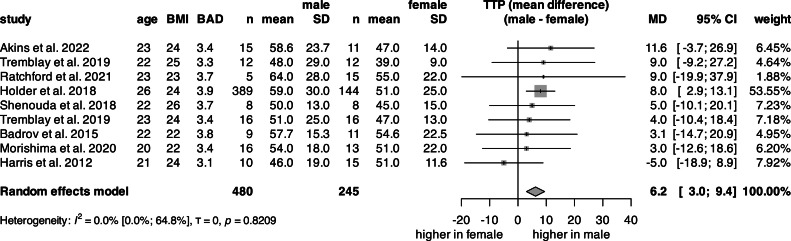
**Forest plot of the mean difference in TTP between sexes.** Vertical lines represent the point estimate, horizontal lines represent the 95 % CI, and squares represent the weight for each study. Diamond represents the pooled mean difference in TTP between sexes. (BMI: body mass index, BAD: baseline artery diameter, n: count, SD: standard deviation, TTP: time-to-peak, 95 % CI: 95 % confidence interval).

The univariate analysis of WSR (Fig. 6, S13) was conducted on 853 subjects from 13 studies. The total mean WSR was 37,093 for females and 28,956 for males. The pooled total mean WSR was 33,079 (Fig. S13). The mean WSR was significantly lower in males compared to females, with a difference of 3421 units (Fig. 7).

In the multivariate linear regression analysis, 798 (35 % female) subjects were analyzed (Table 1, Fig. S14). The model included sex, mean age, and mean BMI, indicating that only sex was a significant predictor of wall shear rate.

### 4.7 Time-to-peak (TTP)

**Fig. 8:**
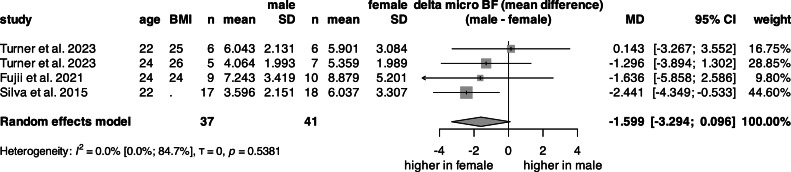
**Forest plot of the mean difference in microΔBF between sexes.** Vertical lines represent the point estimate, horizontal lines represent the 95 % CI, and squares represent the weight for each study. Diamond represents the pooled mean difference in microΔBF. (BMI: body mass index, n: count, SD: standard deviation, microΔBF: relative change in blood flow in microcirculation, 95 % CI: 95 % confidence interval).

The univariate analysis of TTP (Fig. 7, S17) was conducted on 725 subjects from 9 articles. The mean TTP for females was 48.1 s and 54.9 s for males. The total mean TTP was 50.9 s (Fig. S17). There was a significant 6.2-second difference between sexes, meaning males needed, on average, 6.2 more seconds to reach peak dilation (Fig. 8).

In the multivariate linear regression, data from 725 subjects (34 % female) were analyzed (Table 1, Fig. S18). The model, which included sex, mean age, and mean BMI, identified sex as the only significant predictor of TTP.

### 4.8 Relative change in blood flow in microcirculation (microΔBF)

A univariate analysis of mean microΔBF (Fig. 8, S20) was performed on data from 78 subjects (52 % female) across three studies. When stratified by sex, the mean microΔBF was 598.4 % in females and 507.5 % in males, with no statistically significant difference between the groups. The pooled mean microΔBF was 557.5 %. Due to the limited number of available studies, multivariate linear regression analysis was not performed.

### 4.9 Risk of bias assessment

The overall risk of bias score was 6.29 (Table S6). The most significant source of bias was question 5 of the tool, which failed to identify our confounding factors (menopausal status, BMI, and the use of contraception).

### 4.10 Publication bias and heterogeneity

After performing a visual evaluation and Egger’s test, and Begg’s test, evidence of possible publication bias for mean BAD, mean FMD, and mean WSR was found. However, when mean differences were assessed, only WSR AUC showed a potential publication bias. For detailed information, see Supplementary Figs. S3–4, S7–8, and S15–16.

## 5 Discussion

The study presented here revealed sex differences in multiple macro- and microvascular markers of vascular reactivity across various countries in a generally healthy, young, non-smoking population.

In the univariate analysis, our results showed that there is a 2 % difference in FMD between healthy females and males under the mean age of 40. However, in the multivariate analysis, when we analyzed the sex-age and sex-BMI interactions’ effect on FMD, we found the disappearance of this significant difference, as only a non-significant female tendency remained for greater flow-mediated dilation. The model suggests that a 1-unit increase in age or BMI completely obscures the effect of sex on FMD before the age of 40, or the onset of obesity. The non-significant difference of 0.9 percentage points, corresponding to a ∼9 % relative difference, however, may be clinically relevant. This suggests that females below the mean age of 40 may exhibit enhanced endothelial function and better vascular health compared to their age-matched male counterparts. As a reduction of 1 % in FMD is associated with an 8 % to 13 % increased CVD risk [30], the results are in alignment with the fact that young women are protected from CVD, and develop disease a decade later than men [53], after menopause [[15], [16], [17], [54]], when their FMD begins to decline and at a faster rate than that observed in men [36].

The articles included used different cuff placement locations. Previous studies have shown that upper arm occlusion causes a significantly greater flow-mediated dilation than forearm or wrist occlusion [55,56]. The nitric oxide synthase inhibitor L-NNMA blocked the FMD response almost completely in lower arm occlusion, but only partially after upper arm occlusion [55,57]. Thus, nitric oxide mediates FMD in lower arm occlusion to a large extent, but additional physiological signaling pathways are also involved in FMD after upper arm occlusion. In the multivariate linear regression, data from 1177 subjects (41 % female) were analyzed (Table 1, Fig. S6). As only one study with upper-arm occlusion provided separate data for the male and female age and BMI, only the forearm studies were included in the multivariate analysis. The model, which included sex, mean age, and mean BMI, indicated that sex had no significant effect on FMD, whereas age and BMI were significant predictors. Although limited to a few articles, their inclusion in the univariate analysis could explain the FMD difference between our outcome and a previous meta-analysis with only forearm occlusion (6.3 % ± 2.2 % for females vs. 6.1 % ± 1.8 % for males) [58].

Several authors have noted that the disparity in FMD between sexes is due to their differences in baseline diameter [9,[59], [60], [61], [62]]. The difference in baseline artery diameter was ∼0.8 mm, or approximately 20 %, greater for males than for females in the articles included (Fig. 3, Table 1). Some studies used BAD as a covariate for assessment of FMD [58,60]. However, including BAD as a continuous variable would be mathematically redundant, as BAD is the denominator in FMD [9], and statistically redundant, as “sex” already contains BAD as a categorical predictor.

Allometric scaling and shear rate normalization of FMD are also proposed methods to address anatomical differences [9]. The statistical model utilizes the logarithmically transformed change in artery diameter as the outcome variable and logarithmically transformed baseline diameter as a covariate [63]. In the study of Tremblay et al. [64], native FMD was 5.39 ± 2 % vs 6.58 ± 1.62 %, allometrically scaled FMD was 6.5 ± 2.1 % vs 5.7 ± 2.1 %, and the shear AUC corrected FMD was 5.6 ± 2.1 % vs 6.2 ± 2.1 % for males vs females, respectively. Interestingly, allometric scaling suggests that males have higher FMD, while corrected quantities reduced the significant difference. On the contrary, Akins et al. [7] found that black women maintained significantly higher FMD than black males, even after allometric scaling or shear adjustment. Harris et al. [65] showed that the shear rate of women was greater in the menstrual cycle, and normalization of FMD for shear rate showed that female FMD remained greater in the follicular phase than the FMD of their male counterparts. These findings underscore the complexity of interpreting normalized FMD results in relation to sex differences.

Similarly to FMD, there was no significant difference in ΔBF between males and females; however, both age and BMI proved to be significant predictors. Interestingly, the uni- and multivariate analyses showed inverse tendencies between the sexes (Fig. 5, Table 1). The studies reported similar BMI for males and females; however, the differences in body size, composition, and fitness were not taken into account. Nishiyama et al. [66] found that after muscle mass was normalized, there was no longer a significant difference in reactive hyperemia between males and females, as female participants had significantly less muscle mass. The oxidative ability of the investigated tissue is also an important factor [2] as females have faster oxygen transfer dynamics compared to males. Alomari et al. [67] demonstrated that short-term handgrip exercise training enhanced the reactive hyperemic response, even in the absence of significant changes in muscle mass, indicating adaptation to local metabolites. However, reactive hyperemia varies across individuals as a function of oxygen desaturation [68]. Therefore, when the ΔBF between sexes is compared, individual differences should be considered; however, these data were not available for the meta-analysis.

Wall Shear Rate (an adequate surrogate measure of shear stress) is a hemodynamic stimulus for FMD [69]. In univariate analysis, a significant difference was found between sexes in WSR until maximum dilation. After the inclusion of covariates for sex, age, and BMI revealed that sex remains a significant predictor, and females had a ∼10 % higher WSR.

However, the traditional Doppler ultrasound method has some uncertainty in WSR measurement [70]. WSR was estimated from flow velocity in the studies included [7,[64], [65], [66],[71], [72], [73], [74], [75], [76], [77], [78], [79]]. Furthermore, regardless of whether the shear rate is calculated or measured using more advanced Doppler methods, WSR explains only 14 - 31 % of the variance in the FMD [70,80]. WSR is also dependent on BAD, as smaller arteries generate more shear [[30], [73], [81]]. This aligns well with our findings, as the female group showed a higher WSR in the regression analysis model. Women may be more dilated in response to stress due to higher estrogen levels [74], which may contribute to a lower incidence of CVD. Other factors, such as dietary habits [65,75,78] and fitness level [74] or pre-training [71] also mediate the shear response and FMD. However, these factors were not investigated due to the lack of individual-level data.

While shear rate is a key driver of the dilation response, the timing of vasodilation is equally essential. Occlusion leads to the accumulation of oxygen debt in the tissue, which is repaid by increased blood flow after cuff release [82], yet the rate of de- and reoxygenation can vary even within a homogeneous population [83]. Unfortunately, none of the included studies reported this parameter. Therefore, TTP was used to indirectly measure oxygen debt. It was shown that peak WSR is reached faster than peak dilation [70], and that NO production lags behind the elevation in WSR [84]. In this meta-analysis, females reached peak vasodilation significantly faster than males, independent of age or BMI. Aizawa et al. [70] showed that peak shear rate occurs well before peak vasodilation, highlighting a delayed vascular response to elevated shear. Beyond time-to-peak (TTP), the recovery time, or the total hyperemic time, which reflects the duration of elevated blood flow, is also a relevant marker [85].

Due to the distinct differences in the regulation of blood flow between the large arteries and microcirculation, we also investigated the effect of occlusion release on microΔBF of the skin. The microΔBF was measured using Laser Doppler analysis, a well-established method for assessing microvascular health [86]. Univariate analysis revealed no significant difference between sexes in skin reactive hyperemia as a percentage increase; however, females tended to have higher hyperemia.

PORH also has a prognostic value for future cardiovascular events. The involved mechanisms include myogenic, nervous, and metabolic pathways, apart from the endothelium-dependent pathway [34]. To date, the mechanism remains poorly understood, and there is currently no consensus on the relative contributions of endothelium-dependent and -independent pathways [87]. PORH assessment on the skin is a sensitive method. It has been demonstrated that, in addition to estrogen and sex-related differences in regulatory pathways [[88], [89], [90], [91], [92], [93]], which may lead to differences between females and males, other modifying factors also influence the hyperemic response, contributing to both population and interindividual variability. These factors include the location and posture of the skin being investigated [92,94], skin thickness and pigmentation [92], ambient temperature [95], and age of subjects [94]. Moreover, cardiac, myogenic, neurogenic, and respiratory effects also influence laser Doppler flowmetry signals [96]. Interestingly, when Fujii et al. [89] compared the reactive hyperemia response between young and old subjects, age was only a significant predictor for TTP, but not for other PORH parameters. The TTP of cutaneous reactive hyperemia has been linked to Ca²⁺-activated K⁺ channels [97], which are also modulated by estrogen. Therefore, a TTP analysis comparing sexes would be of particular interest; however, this could not be performed at the microvascular level in our study.

There are anatomical and functional differences in the cardiovascular system between men and women that may influence FMD and PORH parameters. Autonomic support for arterial blood pressure [98], venous compliance [99], and arterial stiffness [100] is lower in women, and the heart is smaller [101]. In contrast, heart rate and ventricular contractility are higher [102] in women to compensate for differences in cardiac output. Blood viscosity in males is generally higher compared to females, which may be attributed to hormonal influences, as well as other factors such as BMI and triglyceride levels [103]. Differences in blood viscosity at any age can affect blood flow, shear rate, and, consequently, FMD [104]. The difference increases with age [105].

In addition to anatomical differences, hormonal differences exist between sexes: females generally have higher estrogen and lower testosterone levels than males [[106], [107], [108]]. However, hormonal levels change with age, and these differences are attenuated, particularly after 40–45 years [[106], [107], [108]]. FMD declines with age in both sexes, but the onset and rate of decrease differ: in men, the reduction begins around 41 years, whereas in women it starts later, around 53 years [36]. In females, this age-related decline accelerates during menopause [37], leading to a faster reduction compared to males [38]. In females, menopausal symptoms typically occur between 40 and 50 years of age, with estrogen levels decreasing by 67 % by the first two years and by an additional 18 % by the first 6 years of menopause [109], whereas in males, testosterone levels decline by about 1 % per year [110]. Although hormonal differences persist throughout life, they become smaller with age. Suppose higher FMD in young females contributes to reduced CVD risk due to higher estrogen [111]. In that case, it is plausible that the convergence of hormonal levels with age partly explains the more similar CVD risk between older females and males [112].

### 5.1 Strengths and limitations

This meta-analysis included 51 two-arm studies with over 2000 participants from multiple countries. All studies applied a rigorous methodology. FMD protocols were comparable across studies, and the site of vascular occlusion was consistently controlled, enhancing methodological consistency. Due to our strict criteria for eligibility for multivariate analyses, although we could only include a portion of all studies, this increases their accuracy and power.

The primary limitation of our analysis is that we relied on the means and standard deviations of studies, rather than individual-level data for age and BMI, which may have reduced the precision of our predictive models.

All subjects included in the meta-analyses were non-smokers and were deemed healthy by the original authors; however, exact laboratory parameters were not always provided, thus we could not control their effect. Menopausal transition has a dramatic effect on female FMD [37]. We targeted pre-menopausal women in our study, and we collected articles that either stated that female participants were pre-menopausal or used a female population with a mean age of 40 or less. While hormonal levels were not measured in the included studies, most of them timed their FMD tests during the early follicular phase of the female cycle, similar to a recent FMD guideline [30]. However, a new meta-analysis found that the menstrual cycle has only a small effect on macrovascular endothelial function during the late follicular phase, but essentially has no effect in the luteal phase [113], thus those articles that did not provide the cycle stage of females should not make our findings less valuable.

The included studies differed in the site of occlusion, with some performing forearm and others upper-arm occlusion. We could not include enough studies with upper arm occlusion in the multivariate analysis for FMD; thus, we could not conclude its effect on the extent of FMD. There were also insufficient studies to perform a comparison or subgroup analysis for dBF, WSR, and TTP, which further limits our confidence in conclusions regarding the effect of occlusion site for these parameters.

The small number of studies prevented conducting multivariate regression analysis for skin reactive hyperemia (i.e., microcirculation). Still, the population in the articles included had a narrow age and BMI range.

Another limitation is the lack of racial or ethnic data, which - despite the inclusion of studies from diverse countries - could not be reliably inferred from study location and likely contributed to the observed variability.

### 5.2 Implications for practice and research

This meta-analysis has translational value [114,115], which underscores the importance of accounting for sex differences in both research and clinical settings. In clinical practice, our findings emphasize the importance of sex-specific vascular responses, particularly in conditions that affect endothelial function.

Despite long-standing recommendations, women remain underrepresented in cardiovascular research, comprising <38.2 % of participants in 740 trials reviewed between 2010 and 2017 [116], largely due to concerns about hormonal variability and childbearing potential [[117], [118], [119], [120]]. In research, we strongly recommend that trials ensure balanced sex representation to improve relevance and applicability.

For vascular reactivity studies, we encourage adherence to established methodological guidelines, such as those by Celermajer et al. [28] and subsequent consensus statements [30,96], which help ensure consistency in FMD and laser Doppler flowmetry protocols. We also advise researchers to report BAD, absolute and relative change in diameter, and ΔBF, and avoid including both “sex” and “BAD” in the same regression model, as BAD is a sex-dependent parameter.

## 6 Conclusion

This meta-analysis demonstrates that healthy females below the mean age of 40 exhibit significantly greater flow-mediated dilation (FMD) and faster vasodilation responses than their male counterparts, suggesting superior endothelial function and vascular health. However, when adjusting for age and BMI, the effect of sex is completely obscured, supporting the notion of inherent physiological disparities in vascular reactivity. While unadjusted differences in baseline artery diameter partially explain FMD variation, normalization techniques yield inconsistent results, underscoring the complexity of interpreting sex effects. No significant sex differences were found in reactive hyperemia (ΔBF) or microvascular PORH, though trends favored higher responses in females. A faster time-to-peak dilation in females may reflect estrogen-driven modulation of vascular tone. Wall shear rate (WSR) was slightly higher in females when covariates were considered, providing a further nuance to the observed FMD associations. These findings, however, should not be extrapolated to the general population across all age groups, as we have not studied children, adolescents, and populations above the mean age of forty. Despite methodological heterogeneity, findings were robust across diverse populations and occlusion protocols. Future studies should ensure balanced sex representation, report relevant hemodynamic parameters, and adopt standardized vascular assessment protocols to clarify mechanisms underlying sex-specific cardiovascular risk.

## 7 Author declaration

We wish to confirm that there are no known conflicts of interest associated with this publication and there has been no significant financial support for this work that could have influenced its outcome.

We confirm that the manuscript has been read and approved by all named authors and that there are no other persons who satisfied the criteria for authorship but are not listed. We further confirm that the order of authors listed in the manuscript has been approved by all of us.

We confirm that we have given due consideration to the protection of intellectual property associated with this work and that there are no impediments to publication, including the timing of publication, with respect to intellectual property. In so doing we confirm that we have followed the regulations of our institutions concerning intellectual property.

We understand that the Corresponding Author is the sole contact for the Editorial process (including Editorial Manager and direct communications with the office). He/she is responsible for communicating with the other authors about progress, submissions of revisions and final approval of proofs. We confirm that we have provided a current, correct email address which is accessible by the Corresponding Author.

## Ethical approval

No ethical approval was required for this systematic review with meta-analysis, as all data were already published in peer-reviewed journals. No patients were involved in the design, conduct, or interpretation of our study.

The datasets used in this study can be found in the full-text articles included in the systematic review and meta-analysis.

## Funding

Sponsors had no role in the design, data collection, analysis, interpretation, and manuscript preparation.

## CRediT authorship contribution statement

**Ádám Fekete:** Writing – original draft, Project administration, Methodology, Formal analysis, Conceptualization. **Dániel Horváth:** Writing – review & editing, Validation, Investigation, Data curation. **Patrik Kreuter:** Writing – review & editing, Validation, Investigation, Data curation. **Petrana Martinekova:** Writing – review & editing, Methodology, Conceptualization. **Eszter Ágnes Szalai:** Writing – review & editing, Methodology, Conceptualization. **Gergely Agócs:** Writing – review & editing, Formal analysis, Data curation, Conceptualization. **Péter Sótonyi:** Writing – review & editing. **Gábor Varga:** Writing – review & editing, Conceptualization. **Péter Hegyi:** Writing – review & editing, Conceptualization. **Beáta Kerémi:** Writing – review & editing, Supervision, Conceptualization. **János Vág:** Writing – review & editing, Supervision, Conceptualization.

## Declaration of competing interest

The authors declare that they have no known competing financial interests or personal relationships that could have appeared to influence the work reported in this paper.

## Appendix Supplementary materials

Image, application 1
