# Supplementary material for: Sex differences in macro- and microvascular reactivity – a systematic review and meta-analysis
Source: Am J Prev Cardiol. 2025 Oct 25;24:101335. doi: 10.1016/j.ajpc.2025.101335 (PMC12621484; doi:10.1016/j.ajpc.2025.101335)
Supplement: Supplementary file 1 [file mmc1.docx]

**Title: Sex differences in macro- and microvascular reactivity – a systematic review and meta-analysis**

**Authors**

Ádám Fekete^1,2^, Dániel Horváth^1,3^, Patrik Kreuter^1,4^, Petrana Martinekova^1,5^, Eszter Ágnes Szalai^1,2^, Gergely Agócs^1,6^, Péter Sótonyi^7^, Gábor Varga^1,8^, Péter Hegyi^1,9,10^, Beáta Kerémi^1,2^, János Vág^1,2^

**Affiliations:**

1. Centre for Translational Medicine, Semmelweis University, 1085, Budapest, Baross u 22, Hungary
2. Department of Restorative Dentistry and Endodontics, Semmelweis University, 1088, Budapest, Szentkirályi u. 47, Hungary
3. Department of Oral Diagnostics, Semmelweis University, 1088, Budapest, Szentkirályi u. 47, Hungary
4. Department of Paediatric Dentistry and Orthodontics, Semmelweis University, 1088, Budapest, Szentkirályi u. 47, Hungary
5. Institute for Clinical and Experimental Medicine, Vídeňská 1958, 140 21, Prague, The Czech Republic
6. Department of Biophysics and Radiation Biology, Semmelweis University, 1094 Budapest, Tűzoltó u. 37-47., Hungary
7. Department of Vascular and Endovascular Surgery, Semmelweis University, 1122 Budapest, Városmajor u. 68, Hungary
8. Department of Oral Biology, Semmelweis University, 1089 Budapest, Nagyvárad tér 4, Hungary
9. Institute of Pancreatic Diseases, Semmelweis University, 1083 Budapest, Tömő utca 25-29, Hungary
10. Institute for Translational Medicine, Medical School, University of Pécs, Pécs Szigeti utca 12, Hungary

**Corresponding author: Beáta Kerémi,** DMD, PhD,

Postal address: 1088, Budapest, Szentkirályi u. 47.

Tel.: +36 1 459 1500

E-mail address: keremi.beata@semmelweis.hu

**Supplementary Materials**

**Documentum legends**

**Document S1. Individualized search terms in different databases**

**Document S2. Risk of Bias Assessment Questions according to Checklist For Analytical Cross Sectional Studies, Critical Appraisal tools for use in JBI Systematic Reviews**

**Figures**

**Figure S1. Forest plot of mean BAD, stratified by sex.**

**Figure S2. Multivariate linear regression analysis of mean BAD in relation to age and BMI, stratified by sex (blue: male; red: female**).

**Figure S3. Funnel plot for mean BAD, stratified by sex (blue: male; red: female**).

**Figure S4. Funnel plot for the mean difference in BAD between sexes.**

**Figure S5.** **Forest plot of mean FMD, stratified by sex.**

**Figure S6.** **Multivariate linear regression analysis of mean FMD, in relation to age and BMI, stratified by sex (blue: male; red: female).**

**Figure S7. Funnel plot for mean FMD, stratified by sex (blue: male; red: female**).

**Figure S8. Funnel plot for the mean difference in FMD between sexes.**

**Figure S9.** **Forest plot of mean ΔBF, stratified by sex.**

**Figure S10.** **Multivariate linear regression analysis of the mean ΔBF in relation to age and BMI, stratified by sex (blue: male; red: female).**

**Figure S11. Funnel plot for mean ΔBF, stratified by sex (blue: male; red: female**).

**Figure S12. Funnel plot for the mean difference in ΔBF between sexes.**

**Figure S13.** **Forest plot of mean WSR, stratified by sex.**

**Figure S14.** **Multivariate linear regression analysis of mean WSR in relation to age and BMI, stratified by sex (blue: male; red: female).**

**Figure S15. Funnel plot for mean WSR, stratified by sex (blue: male; red: female**).

**Figure S16. Funnel plot for the mean difference in WSR between sexes.**

**Figure S17** **Forest plot of mean TTP, stratified by sex.**

**Figure S18. Multivariate linear regression analysis of mean TTP in relation to age and BMI, stratified by sex (blue: male; red: female).**

**Figure S19. Funnel plot for mean TTP, stratified by sex (blue: male; red: female**).

**Figure S20. Funnel plot for mean difference in TTP between sexes.**

**Figure S21.** **Forest plot of mean microΔBF, stratified by sex.**

**Figure S22. Funnel plot for mean microΔBF, stratified by sex (blue: male; red: female**).

**Figure S23. Funnel plot for mean difference in microΔBF between sexes.**

**Tables**

**Table S1. PRISMA 2020 for Abstract Checklist.**

**Table S2. PRISMA checklist.**

**Table S3. List of articles that were left out from the meta-analysis.**

**Table S4. Main characteristics of the included studies in the systematic review and meta-analysis.**

**Table S5. Main characteristics of the included studies in the systematic review and meta-analysis.**

**Table S6. Tabular display of Risk of bias assessment with** **Checklist For Analytical Cross Sectional Studies, Critical Appraisal tools for use in JBI Systematic Reviews.**

**Document S1. Individualized search terms in different databases**

**MEDLINE**

(microcirculation* OR microvascul* OR ("blood" AND "flow") OR FMD OR (("flow" AND "mediated") OR ("shear" AND "mediated") OR ("conductive") OR ("endothelium" AND "dependent") AND (vasodil* OR dilat*)) OR (arter* AND "reactivity") OR ("vascular" AND "reactivity") OR ("shear" AND "stress") OR ("shear" AND "rate") OR reperfus* OR ("reactive" AND hyper*) OR ("vascular" AND "function") OR "occlusive" OR "PORH" OR ischem*) AND (brachial OR cutan* OR cutis) AND (ultrasonic OR ultrasound OR doppler OR LDF OR ("laser" AND "speckle")) AND (gender OR (males AND females) OR (woman AND man))

**Embase**

(microcirculation OR microvascul* OR (blood AND flow) OR (flow AND mediated AND vasodil*) OR FMD OR (arterial AND reactivity) OR (blood AND vessel AND reactivity) OR (shear AND mediated AND (vasodil* OR dilat*)) OR (shear AND stress) OR (shear AND rate) OR (conductive AND (vasodil* OR dilat*)) OR reperfusion OR (endothelium-dependent AND (vasodil* OR dilat*)) OR (endothelium AND dependent AND (vasodil* OR dilat*)) OR (reactive AND hyper*) OR (vascular AND function) OR occlusive OR occlusion OR porh OR ischemic OR ischemia OR hyperemia) AND (brachial OR skin OR cutan*) AND (ultrasonic OR ultrasound OR (Doppler AND flowmetry) OR (doppler AND ultrasonography) OR (laser AND doppler) OR LDF OR (laser AND speckle)) AND (sex OR gender OR (male* AND female*) OR (wom?n AND m?n))

**CENTRAL**

(microcirculation OR microvascul* OR (blood AND flow) OR (flow AND mediated AND vasodil*) OR FMD OR (arterial AND reactivity) OR (blood AND vessel AND reactivity) OR (shear AND mediated AND (vasodil* OR dilat*)) OR (shear AND stress) OR (shear AND rate) OR (conductive AND (vasodil* OR dilat*)) OR reperfusion OR (endothelium-dependent AND (vasodil* OR dilat*)) OR (endothelium AND dependent AND (vasodil* OR dilat*)) OR (reactive AND hyper*) OR (vascular AND function) OR occlusive OR occlusion OR porh OR ischemic OR ischemia OR hyperemia) AND (brachial OR skin OR cutan*) AND (ultrasonic OR ultrasound OR (Doppler AND flowmetry) OR (doppler AND ultrasonography) OR (laser AND doppler) OR LDF OR (laser AND speckle)) AND (sex OR gender OR (male AND female) OR (wom?n AND m?n))

**Document S2. Risk of Bias Assessment Questions according to JBI**

## Were the criteria for inclusion in the sample clearly defined?

## Were the study subjects and the setting described in detail?

## Was the exposure measured in a valid and reliable way?

## Were objective, standard criteria used for measurement of the condition?

## Were confounding factors identified?

## Were strategies to deal with confounding factors stated?

##
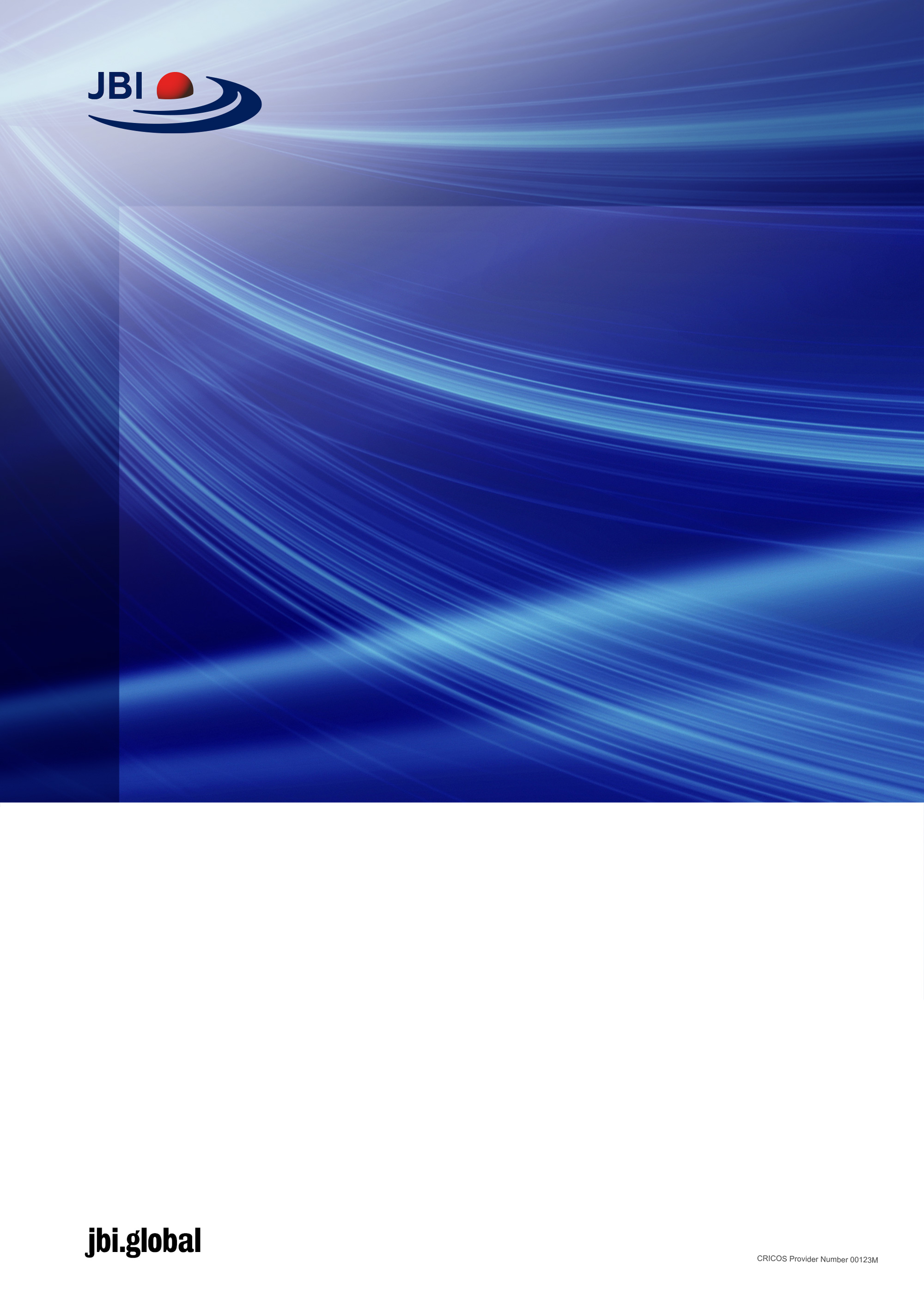
Were the outcomes measured in a valid and reliable way?

## Was appropriate statistical analysis used?

##
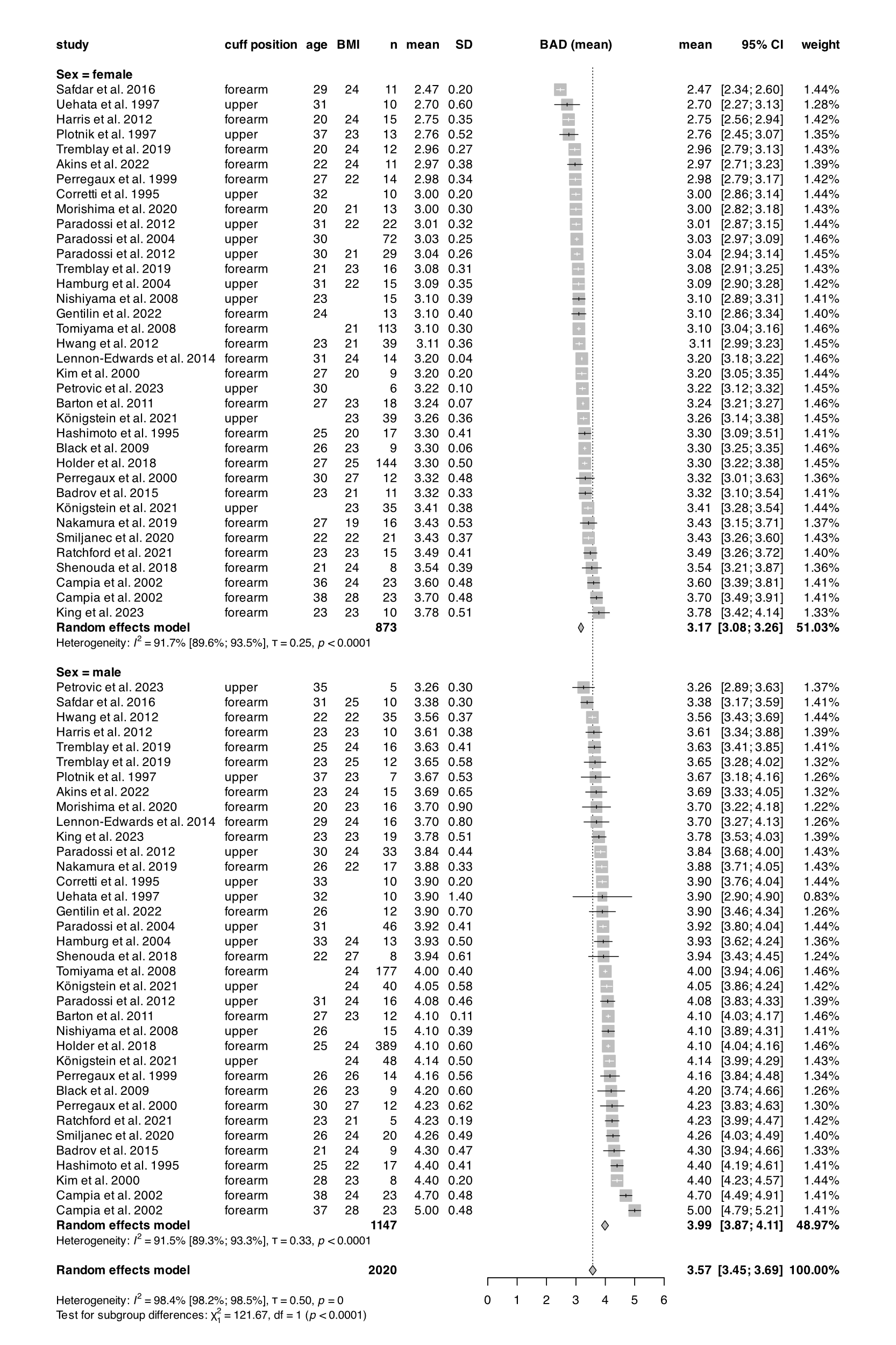


**Figure S1. Forest plot of mean BAD, stratified by sex.** Vertical lines represent the pooled point estimate, horizontal lines represent the 95% CI, and squares represent the weight of each study. Diamonds represent the pooled mean BAD (mm) across the studies. (BMI: body mass index, n: count, SD: standard deviation, BAD: baseline artery diameter, 95% CI: 95% confidence interval)


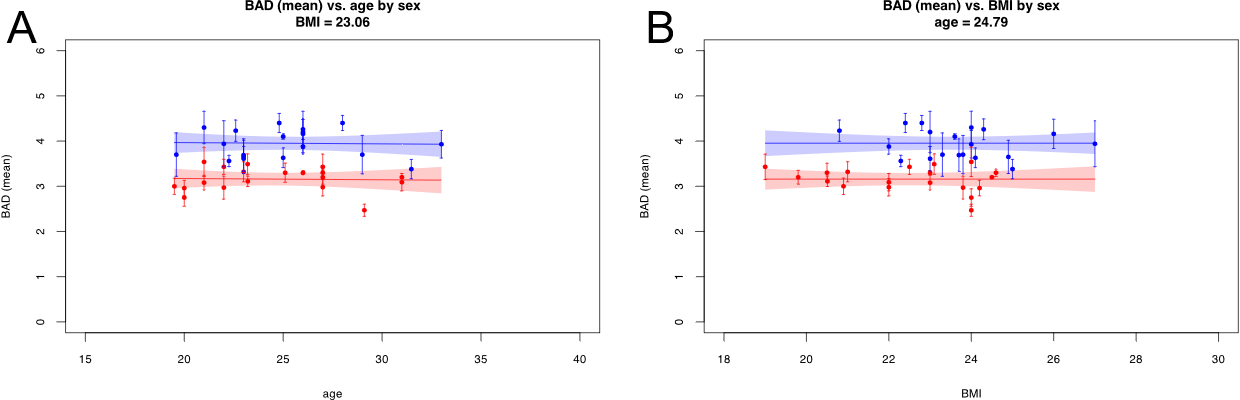


**Figure S2. Multivariate linear regression analysis of mean BAD in relation to age and BMI, stratified by sex (blue: male; red: female**)**.** Figure S2A shows the change of mean BAD (mm) in relation to mean age with fitted regression lines. The BMI is fixed at 23.06. The dots represent the studies' pooled point estimate; the vertical lines represent the 95% CI. Figure S2B shows the change of mean BAD in relation to mean BMI with fitted regression lines. The age is fixed at 24.79. The dots represent the studies' pooled point estimate; the vertical lines represent the 95% CI. (BMI: body mass index, BAD: baseline artery diameter)

**
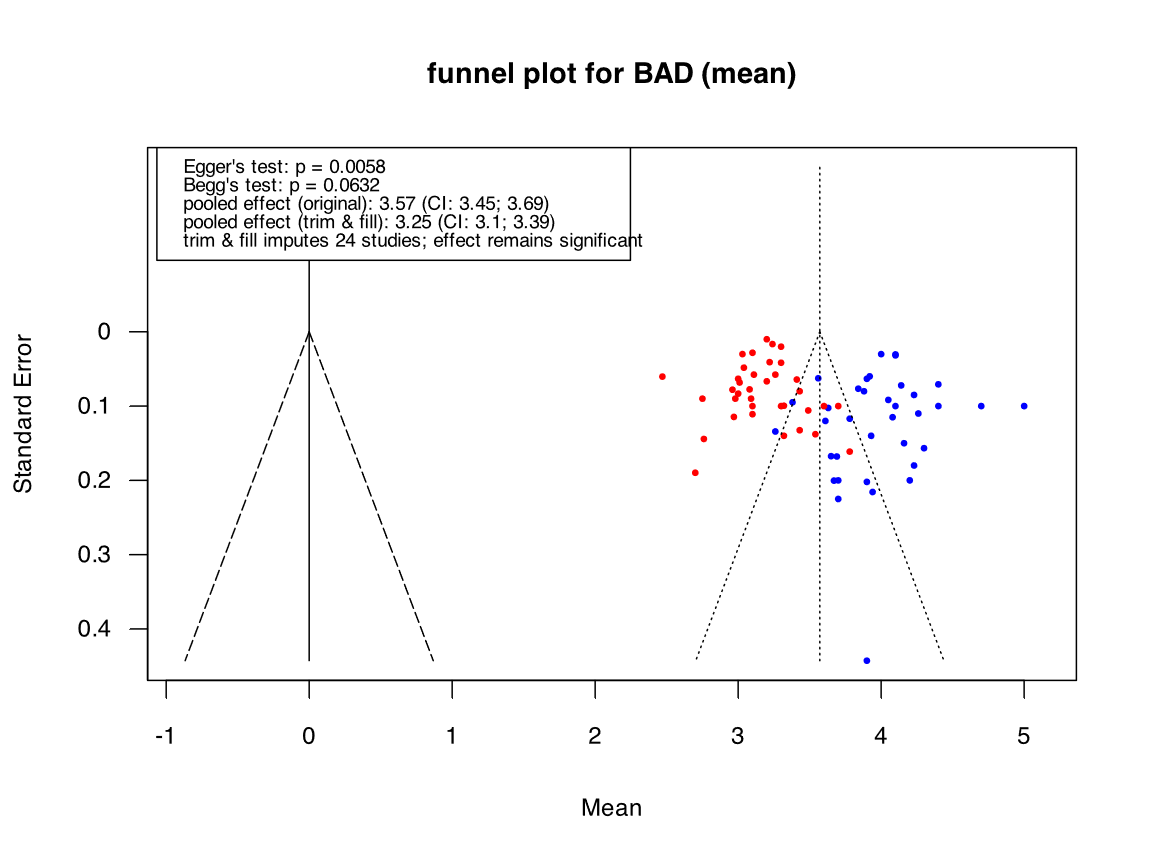
**

**Figure S3. Funnel plot for mean BAD, stratified by sex (blue: male; red: female**). **(**BAD: baseline artery diameter (mm))


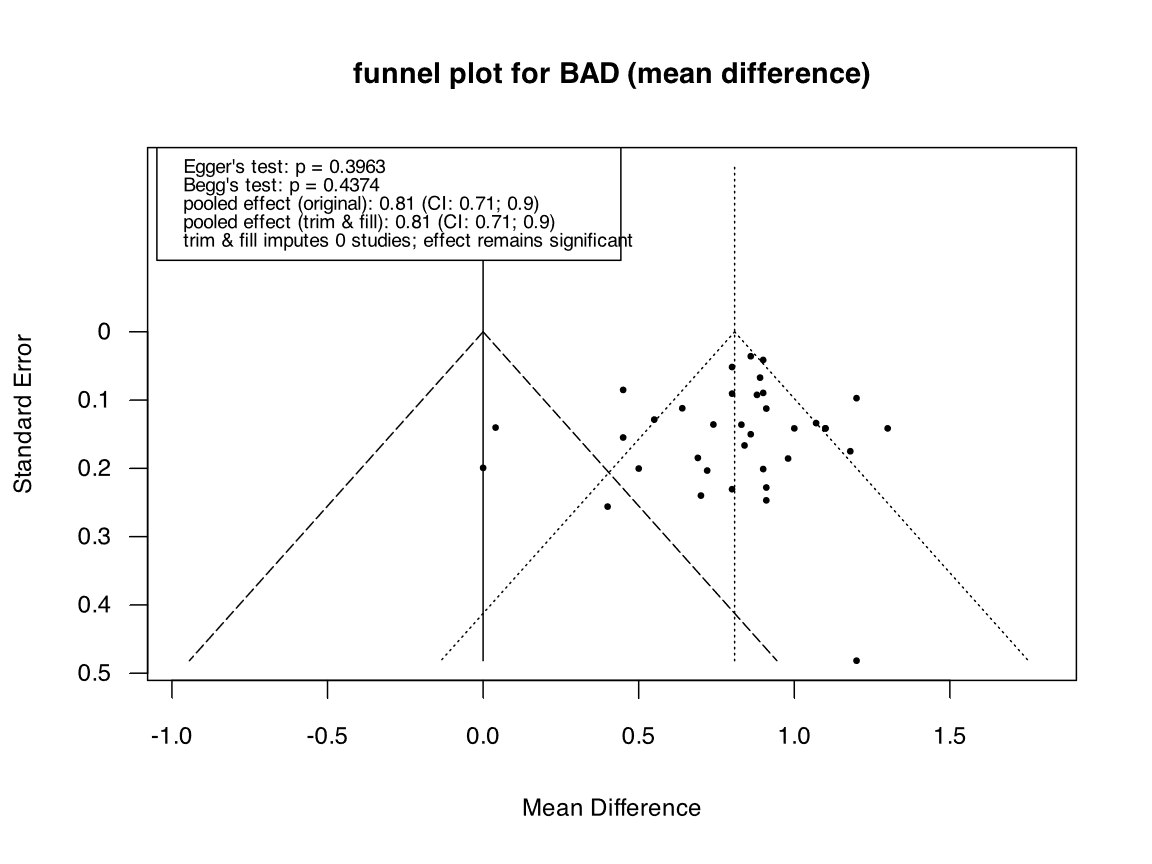


**Figure S4. Funnel plot for the mean difference in BAD between sexes. (**BAD: baseline artery diameter (mm))

**
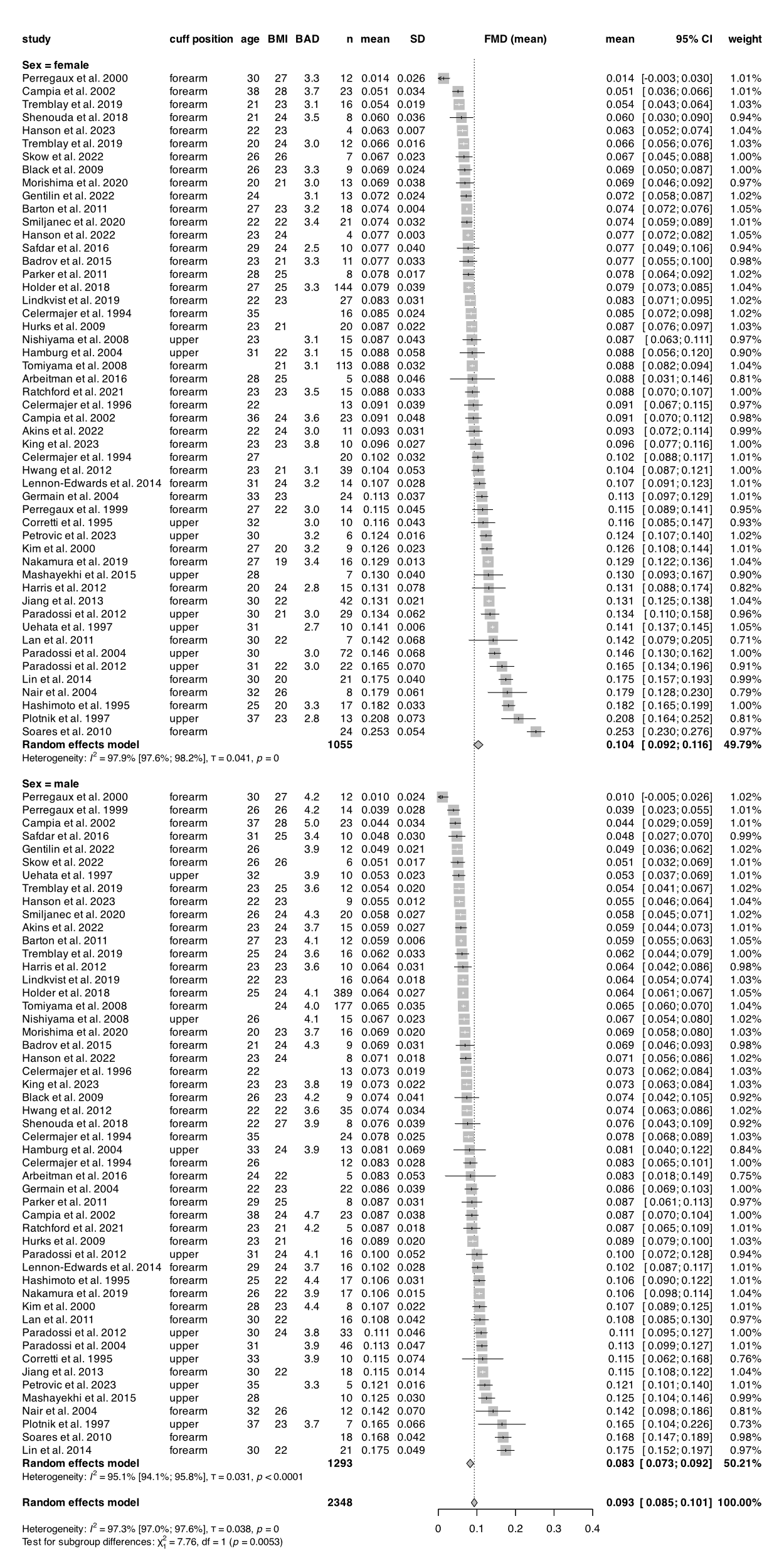
**

**Figure S5.** **Forest plot of mean FMD, stratified by sex.** Vertical lines represent the pooled point estimate, horizontal lines represent the 95% CI, and squares represent the weight of each study. Diamonds represent the pooled mean FMD (%) across the studies. Values are expressed as relative changes. (BMI: body mass index, BAD: baseline artery diameter, n: count, SD: standard deviation, FMD: flow-mediated dilation, 95% CI: 95% confidence interval)

**
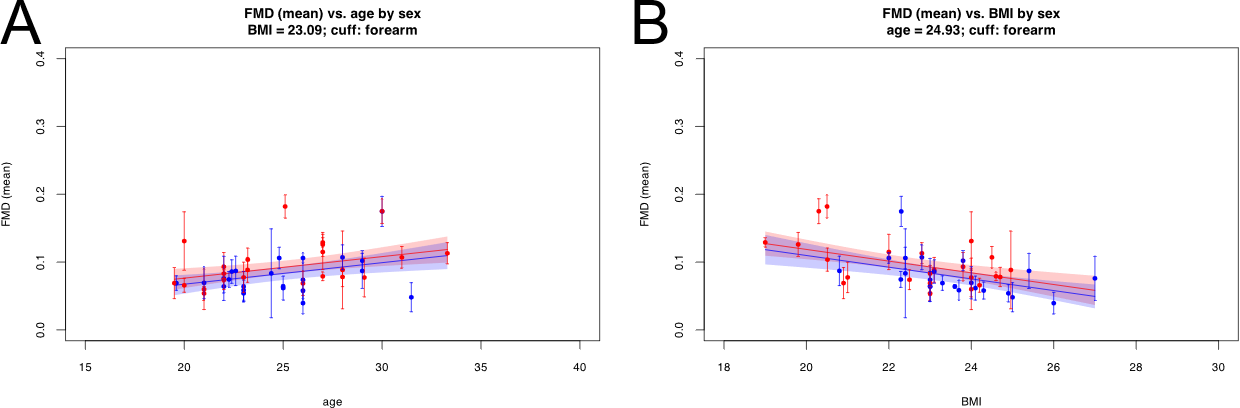
**

**Figure S6.** **Multivariate linear regression analysis of mean FMD in relation to age and BMI, stratified by sex (blue: male; red: female).** Figure S6A shows the change of mean FMD% in relation to mean age, with fitted regression lines. The BMI is fixed at 23.09. The dots represent the mean of each individual study; the vertical lines represent the 95% CI. S6B shows the change of mean FMD% in relation to mean BMI, with fitted regression lines. The age is fixed at 24.93. The dots represent the mean of each individual study; the vertical lines represent the 95% CI.

**
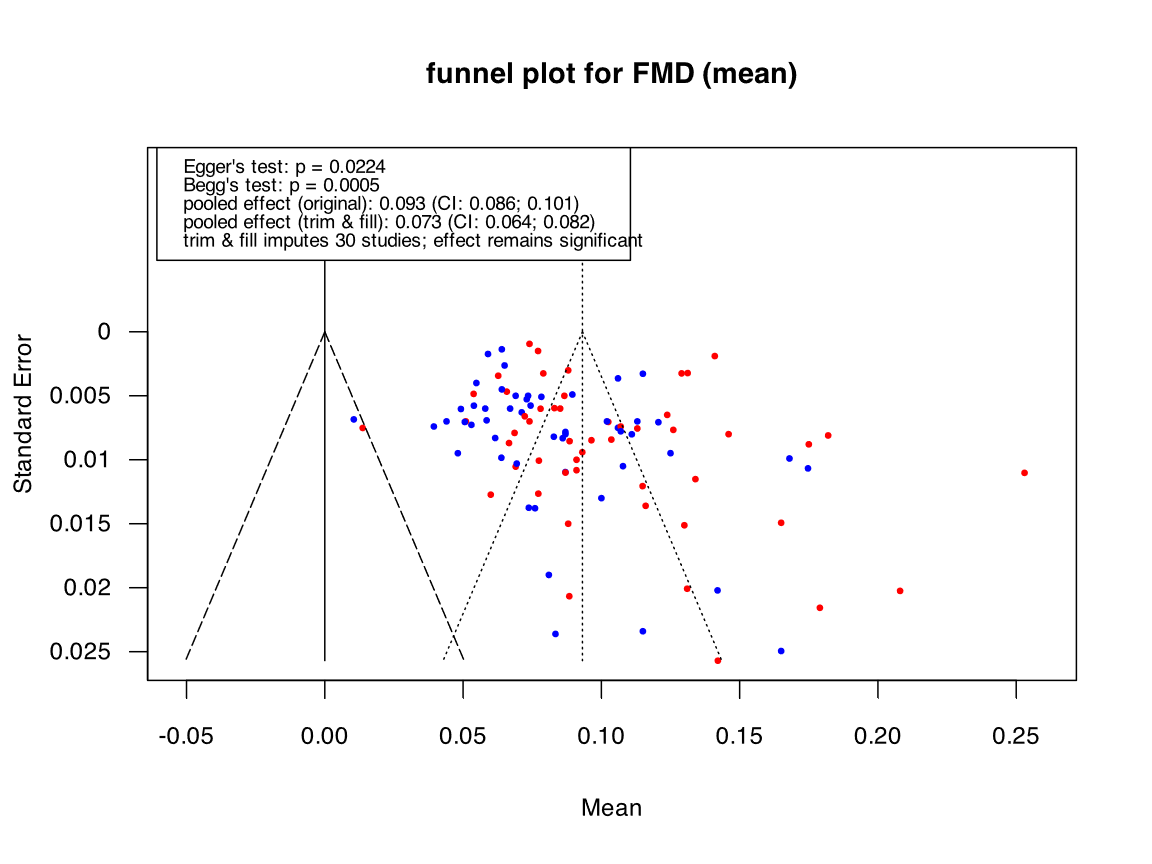
**

**Figure S7. Funnel plot for mean FMD, stratified by sex (blue: male; red: female**). (FMD: flow-mediated dilation (%))

**
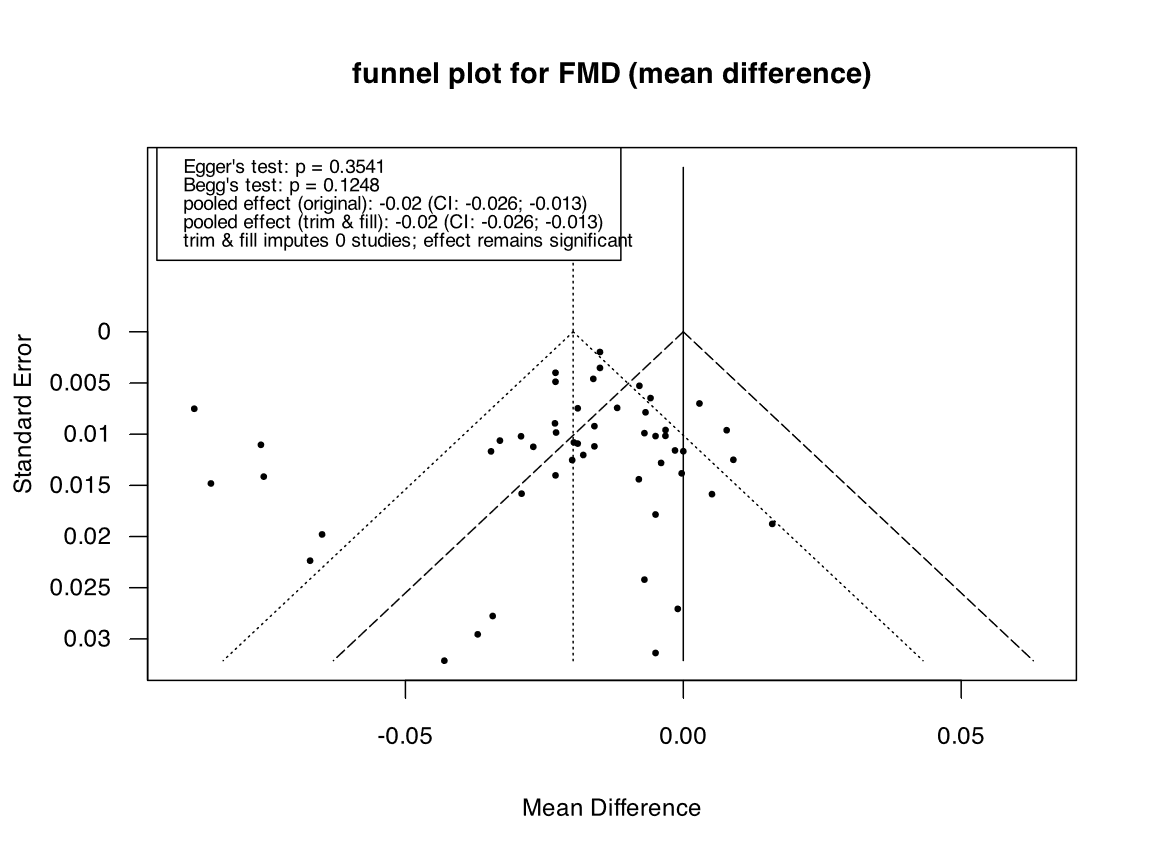
**

**Figure S8. Funnel plot for the mean difference in FMD between sexes.** (FMD: flow-mediated dilation (%))

**
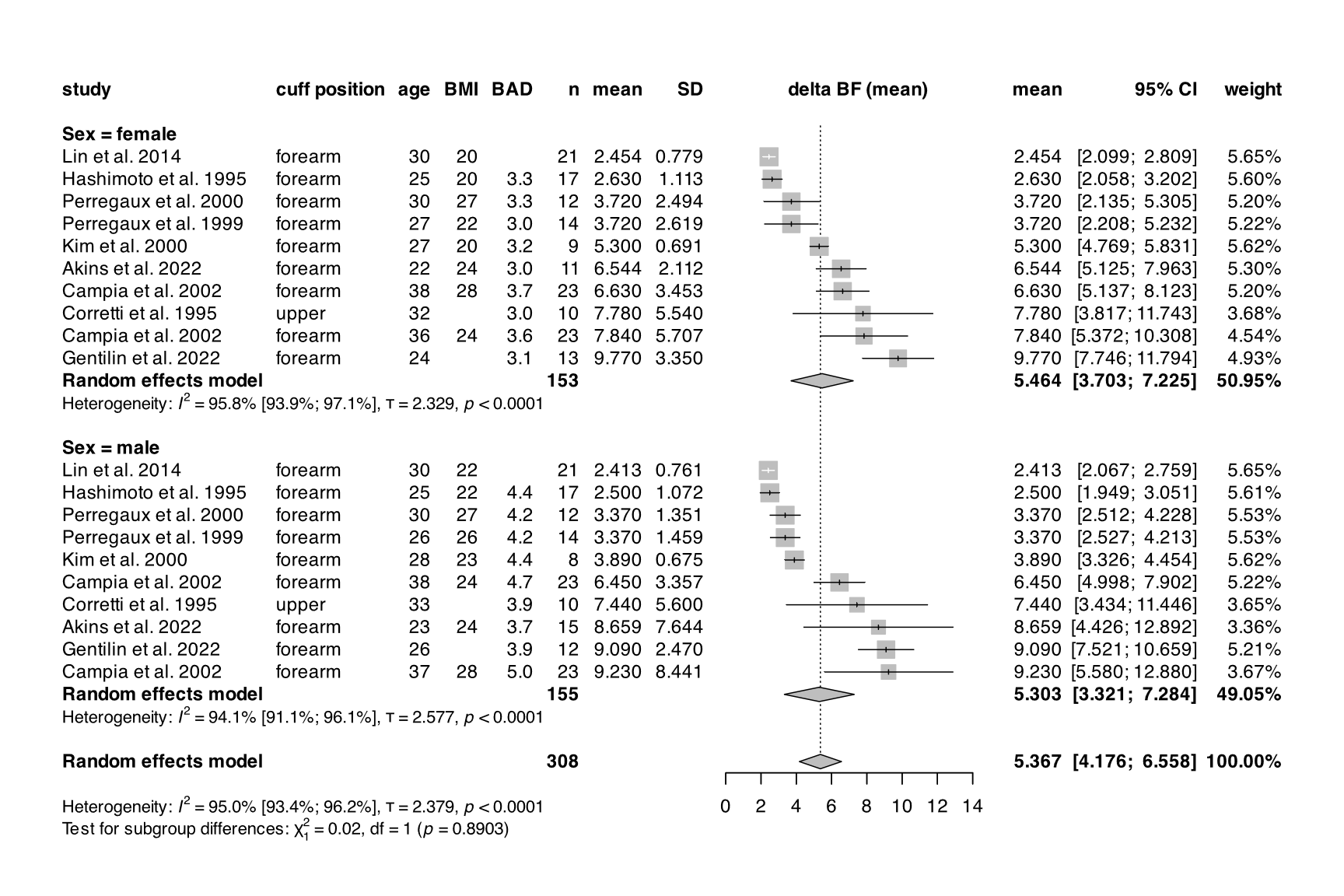
**

**Figure S9.** **Forest plot of mean ΔBF, stratified by sex.** Vertical lines represent the pooled point estimate across sexes; horizontal lines represent the 95% CI, and squares represent the weight of each study. Diamonds represent the pooled mean ΔBF (%) across the studies. (BMI: body mass index, BAD: baseline artery diameter, n: count, SD: standard deviation, ΔBF: relative change in blood flow on the brachial artery, 95% CI: 95% confidence interval)

**
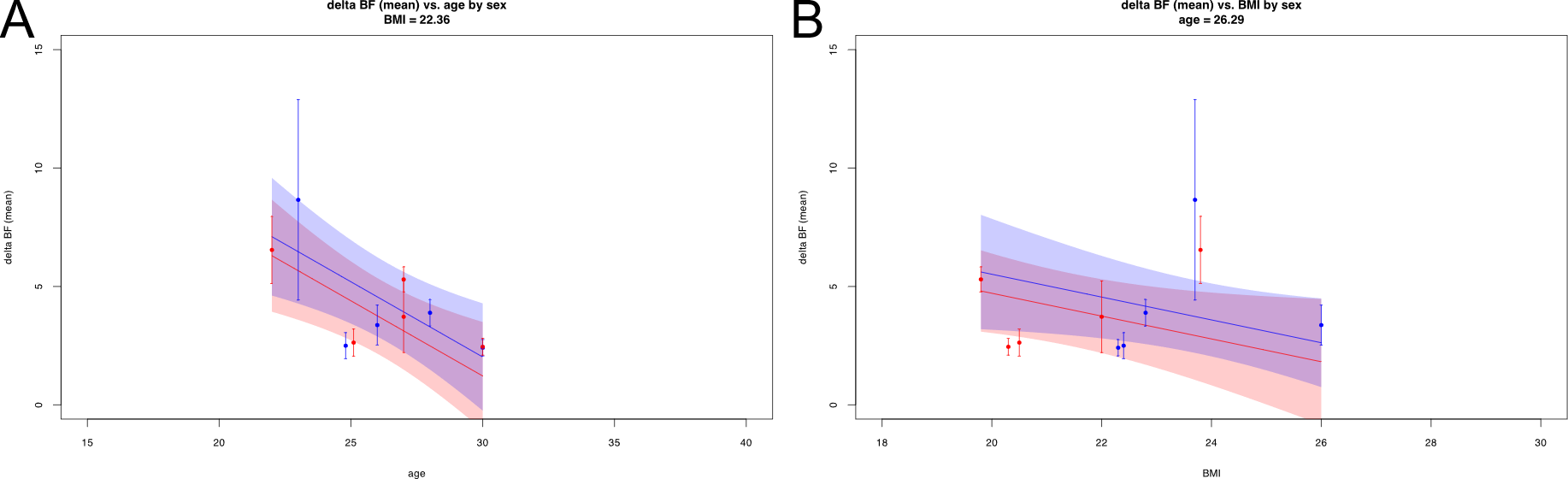
**

**Figure S10.** **Multivariate linear regression analysis of the mean ΔBF, stratified by sex (blue: male; red: female).** Figure S10A shows the ΔBF (%) in relation to mean age with fitted regression lines. The BMI is fixed at 22.36. The dots represent the mean of each individual study; the vertical lines represent the 95% CI. Figure S10B shows the ΔBF (%) in relation to mean BMI with fitted regression lines. The age is fixed at 26.29. The dots represent the mean of each individual study; the vertical lines represent the 95% CI. (BMI: body mass index, ΔBF: relative change in blood flow on the brachial artery)


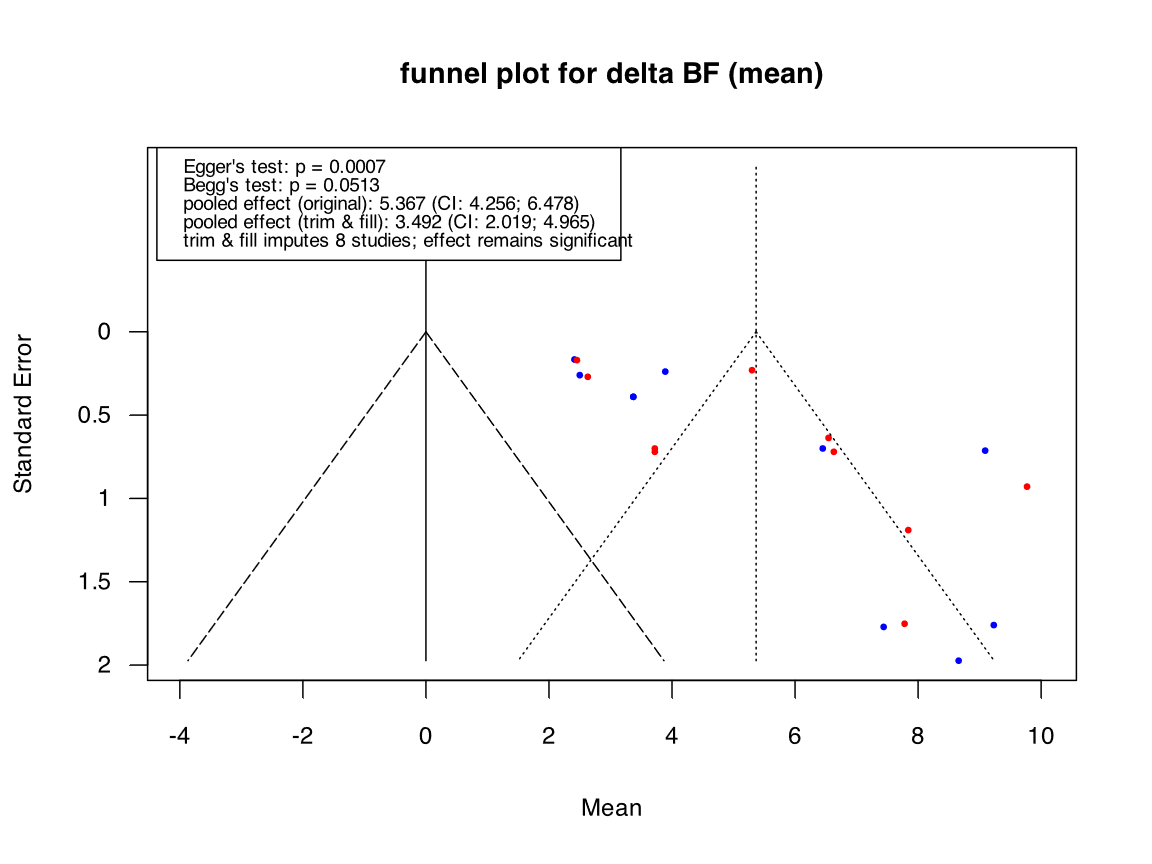


**Figure S11. Funnel plot for mean ΔBF, stratified by sex (blue: male; red: female**). (ΔBF: relative change in blood flow on the brachial artery (%))

**
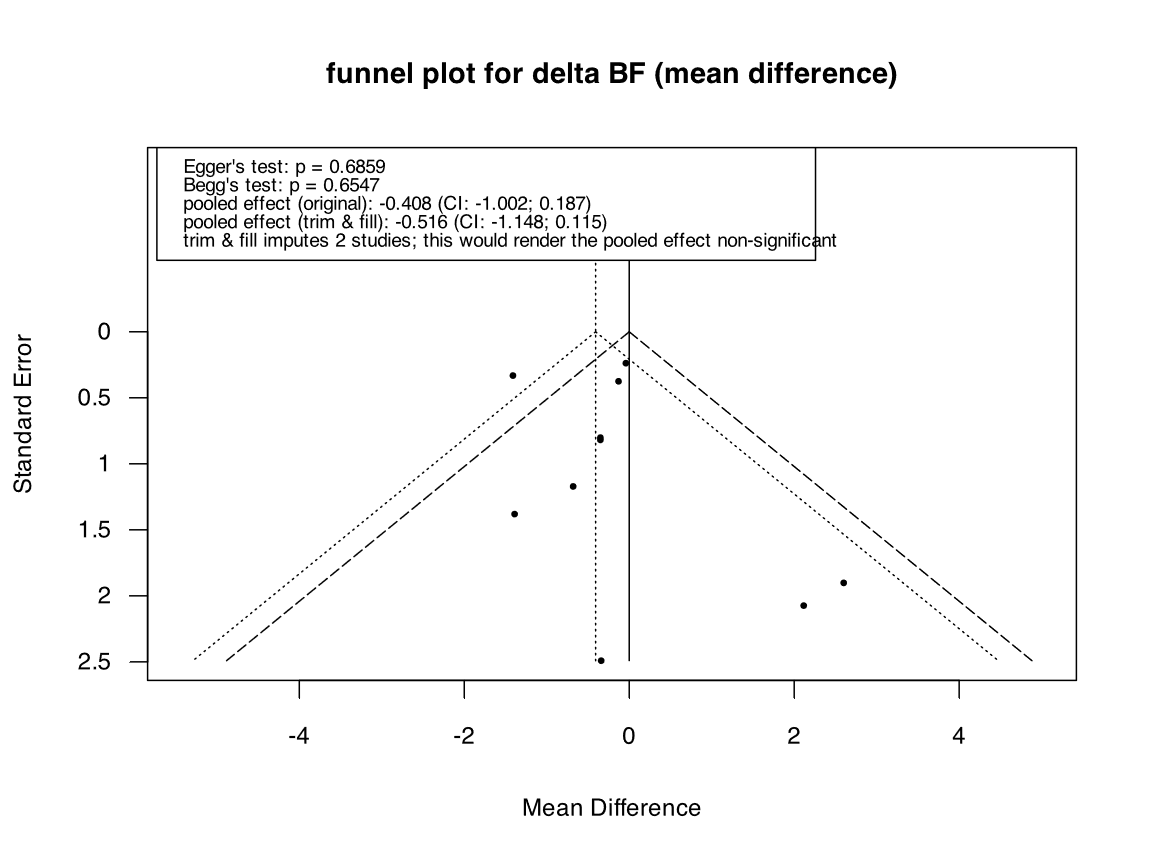
**

**Figure S12. Funnel plot for the mean difference in ΔBF between sexes.** (ΔBF: relative change in blood flow on the brachial artery (%))

**
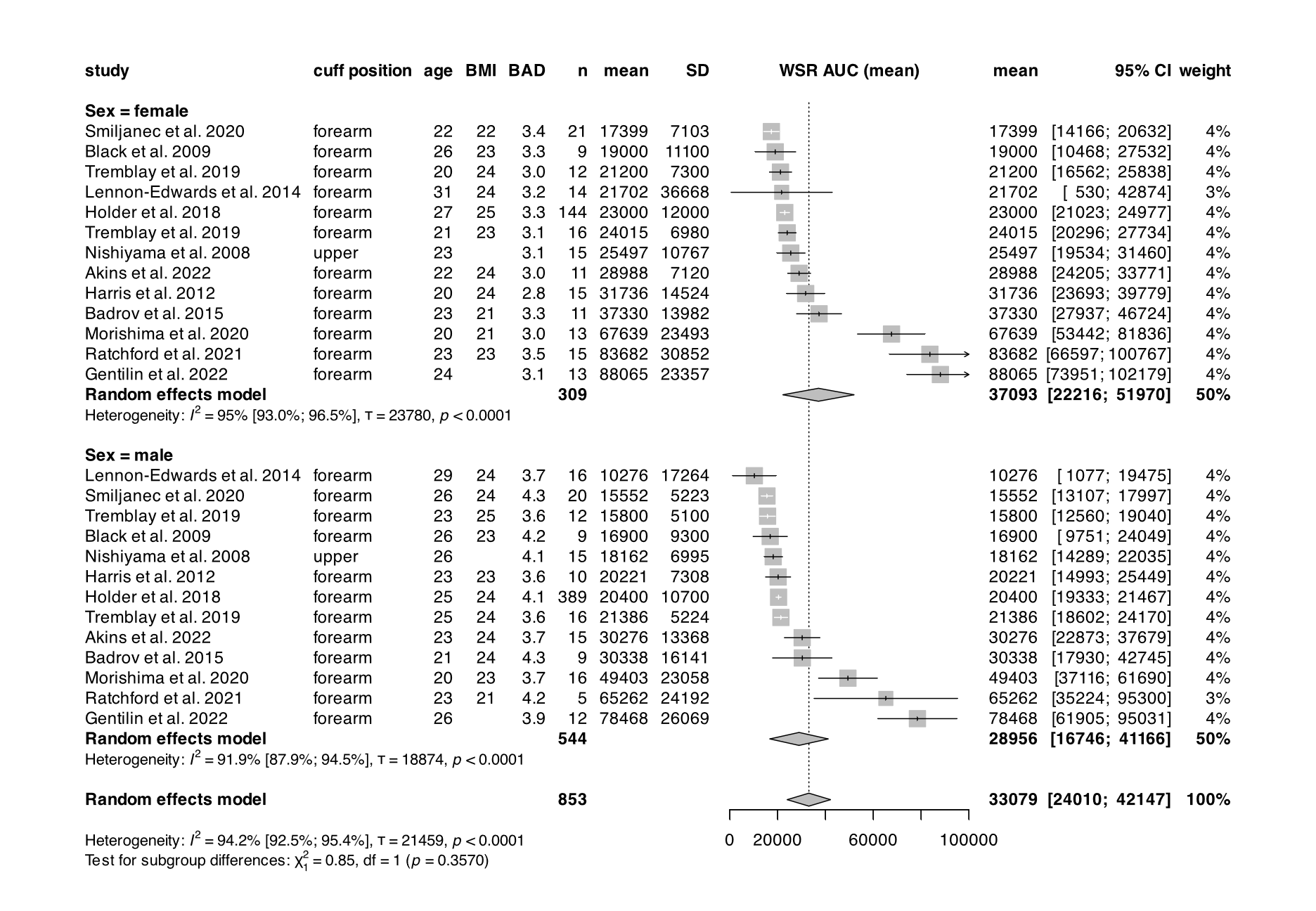
**

**Figure S13.** **Forest plot of mean WSR, stratified by sex.** Vertical lines represent the pooled point estimate across sexes; horizontal lines represent the 95% CI, and squares represent the weight of each study. Diamonds represent the pooled mean WSR across the studies. (BMI: body mass index, BAD: baseline artery diameter, n: count, SD: standard deviation, WSR AUC: wall shear rate area under the curve, 95% CI: 95% confidence interval)


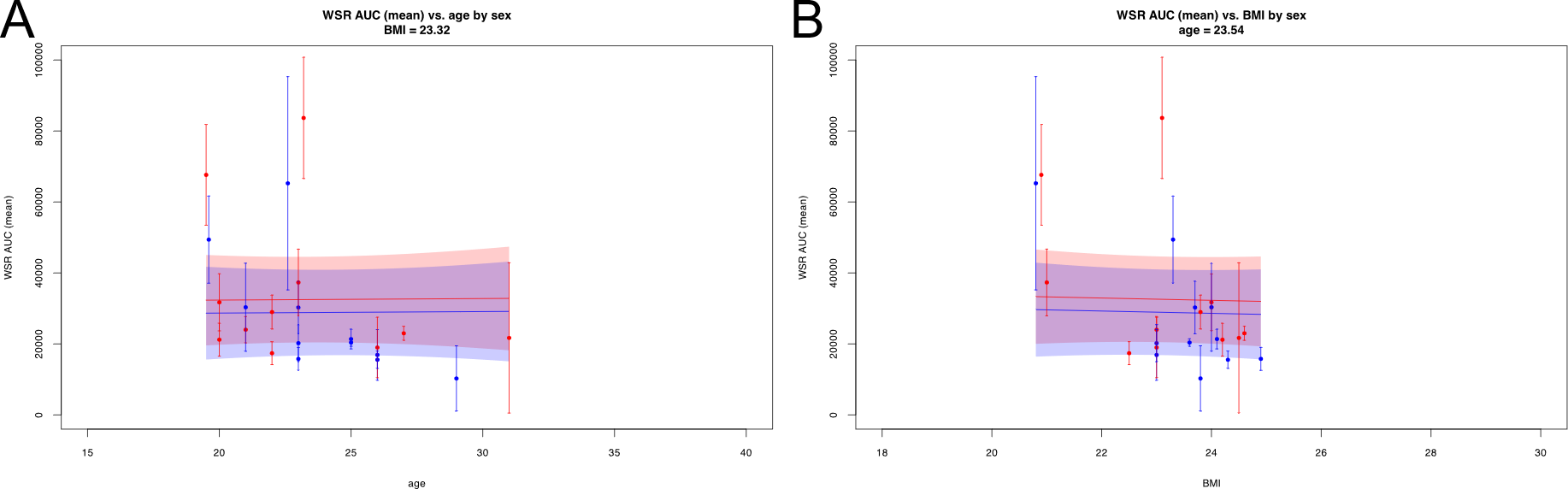


**Figure S14.** **Multivariate linear regression analysis of mean WSR, stratified by sex (blue: male; red: female).** Figure S14A shows the change of mean WSR in relation to mean age with fitted regression lines. The BMI is fixed at 23.32. The dots represent the studies' pooled point estimate; the vertical lines represent the 95% CI. Figure S14B shows the change of mean WSR in relation to mean BMI with fitted regression lines. The age is fixed at 23.54. The dots represent the studies pooled point estimate, the vertical lines represent the 95% CI. (BMI: body mass index, WSR AUC: wall shear rate area under the curve)


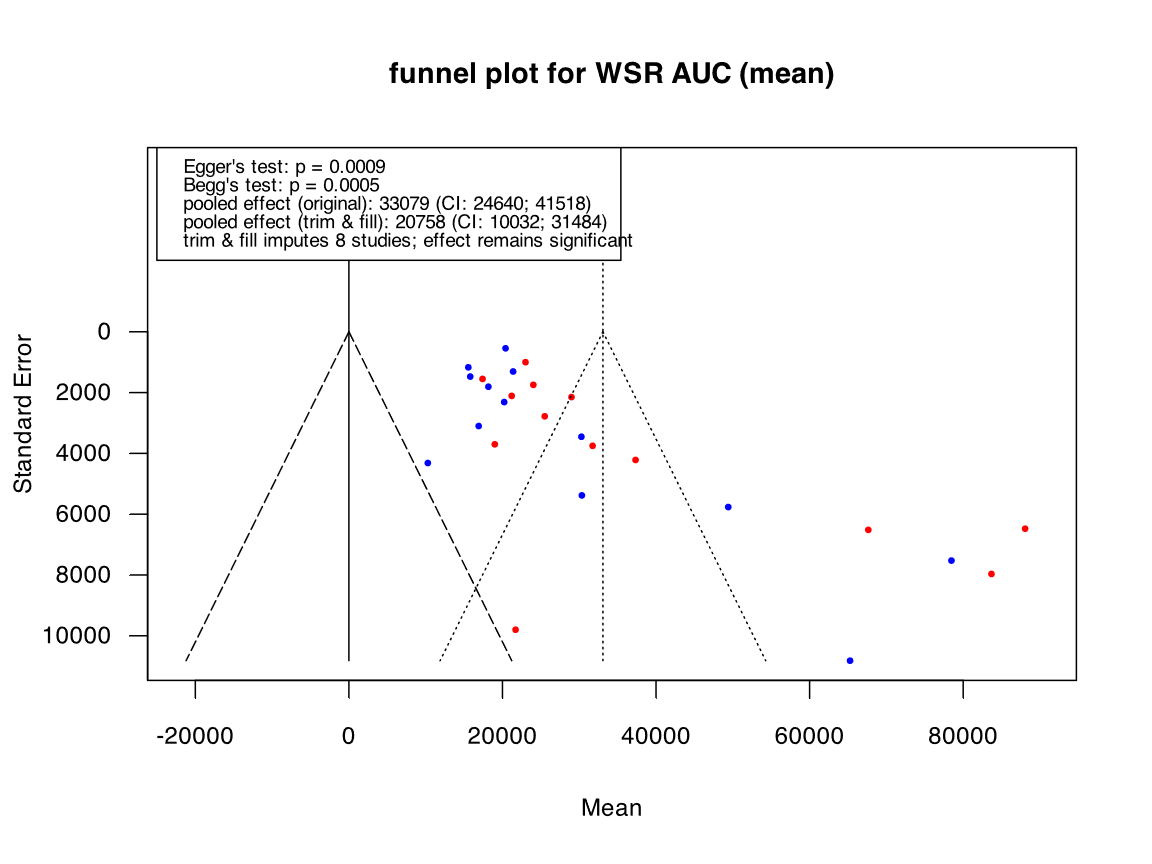


**Figure S15. Funnel plot for mean WSR, stratified by sex (blue: male; red: female**). (WSR AUC: wall shear rate area under the curve)


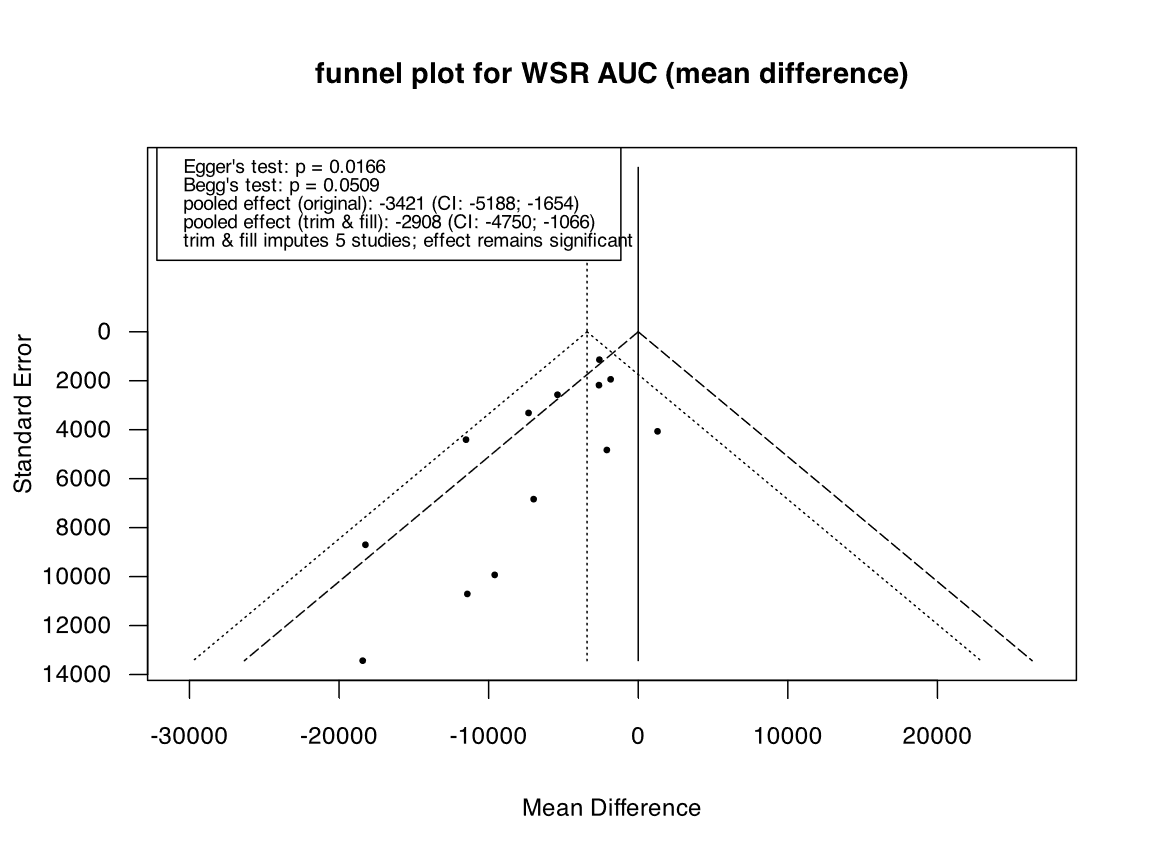


**Figure S16. Funnel plot for mean difference in WSR between sexes.** (WSR AUC: wall shear rate area under the curve)

**
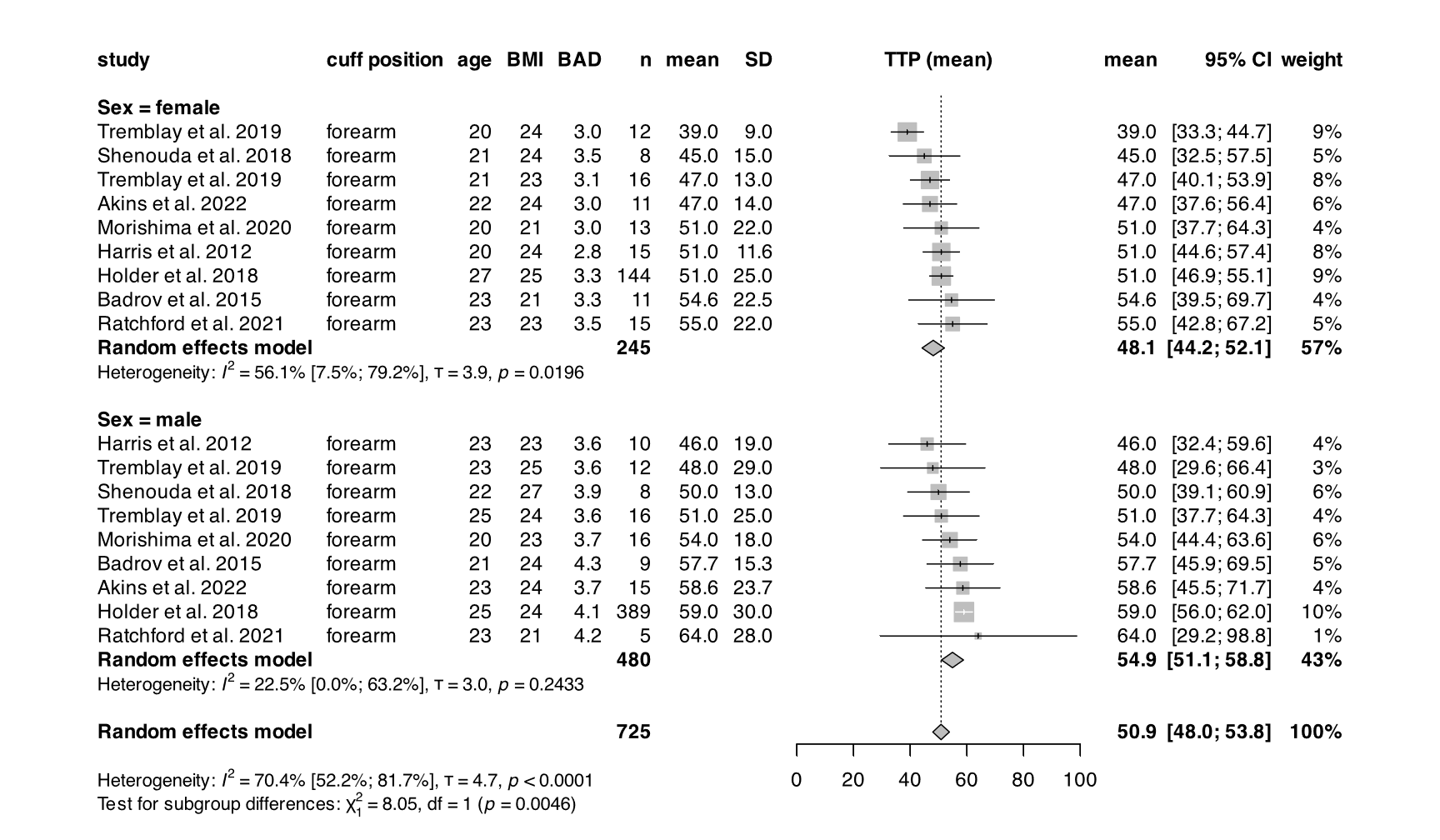
**

**Figure S17.** **Forest plots of mean TTP, stratified by sex.** Vertical lines represent the pooled point estimate across sexes; horizontal lines represent the 95% CI, and squares represent the weight of each study. Diamonds represent the pooled mean TTP (seconds) across the studies. (BMI: body mass index, BAD: baseline artery diameter, n: count, SD: standard deviation, TTP: time-to-peak, 95% CI: 95% confidence interval)


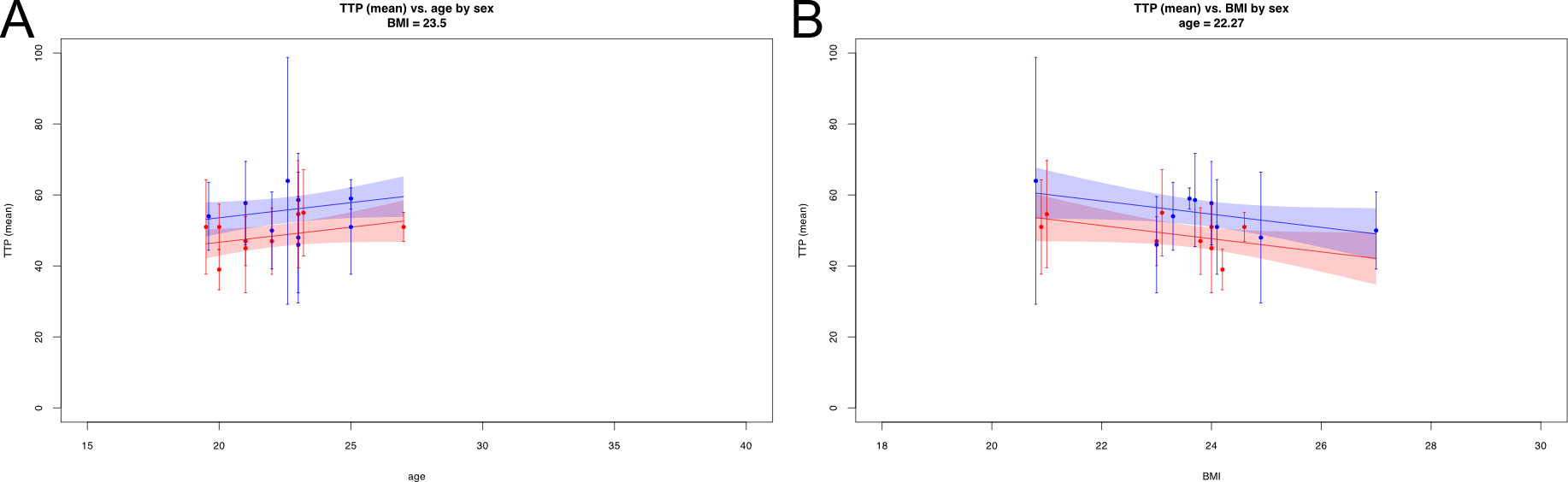


**Figure S18. Multivariate linear regression analysis of mean TTP, stratified by sex (blue: male; red: female).** Figure S18A shows the change of mean TTP (seconds) in relation to mean age with fitted regression lines. The BMI is fixed at 23.5. The dots represent the mean of each individual study; the vertical lines represent the 95% CI. Figure S18B shows the change of mean TTP (seconds) in relation to mean BMI with fitted regression lines. The age is fixed at 22.27. The dots represent the mean of each individual study; the vertical lines represent the 95% CI. (BMI: body mass index, TTP: time-to-peak (seconds))


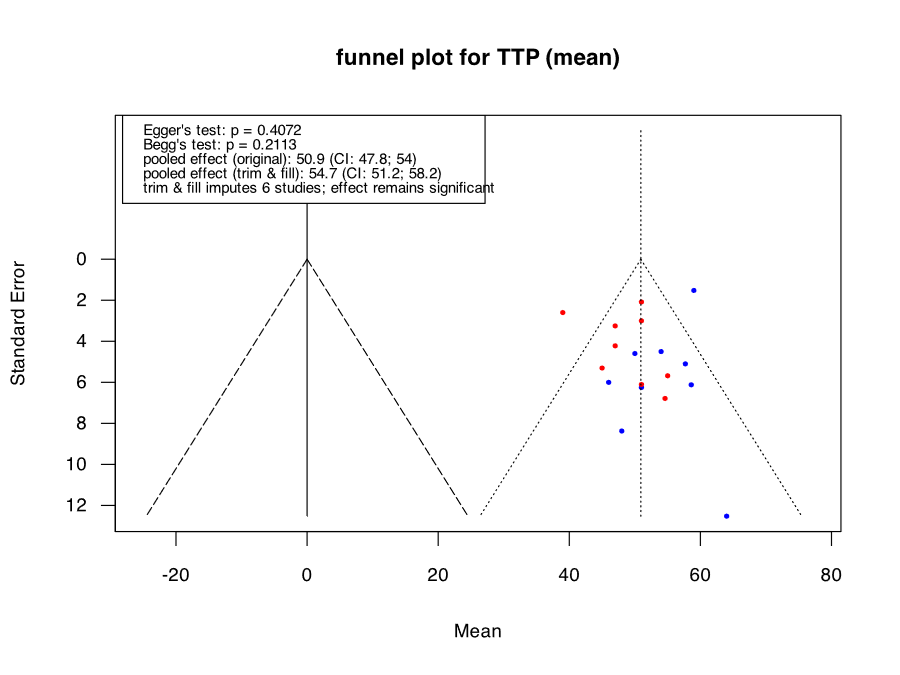


**Figure S19. Funnel plot for mean TTP, stratified by sex (blue: male; red: female**). (TTP: time-to-peak)


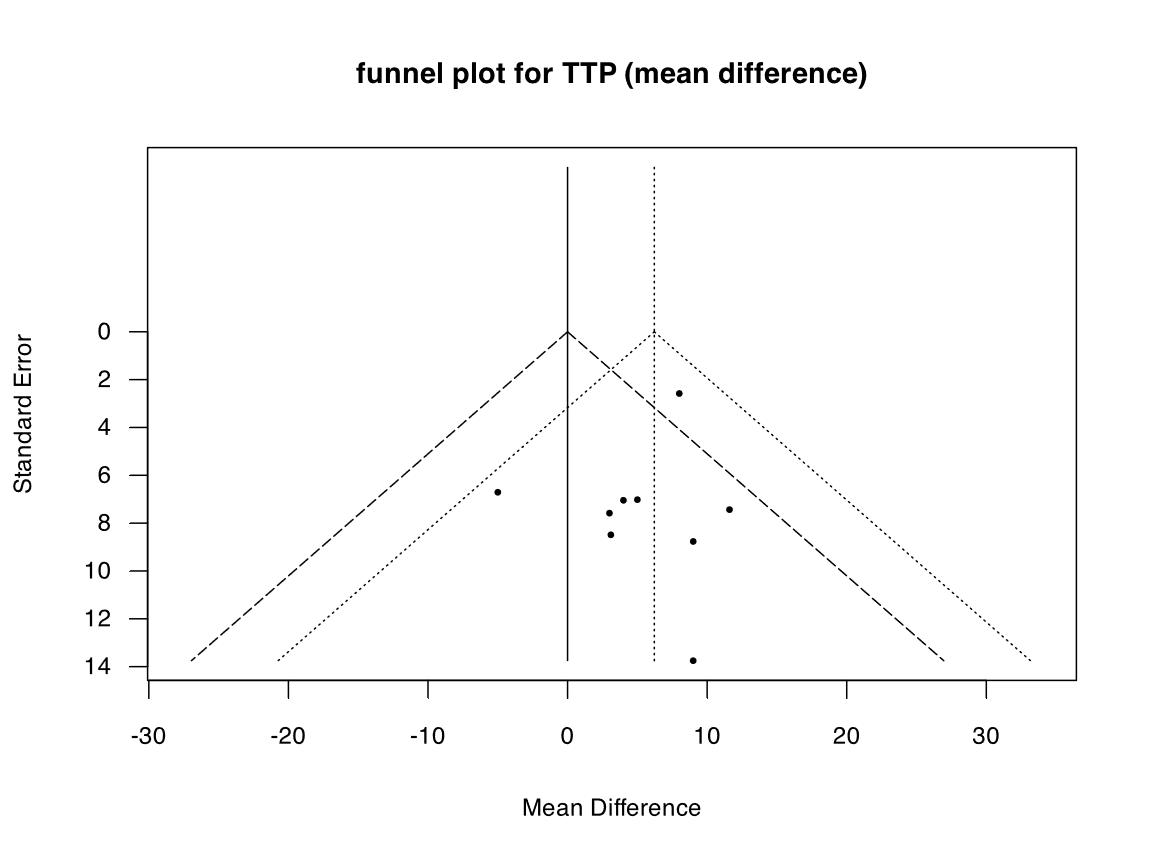


**Figure S20. Funnel plot for mean difference in TTP between sexes.** (TTP: time-to-peak)

**
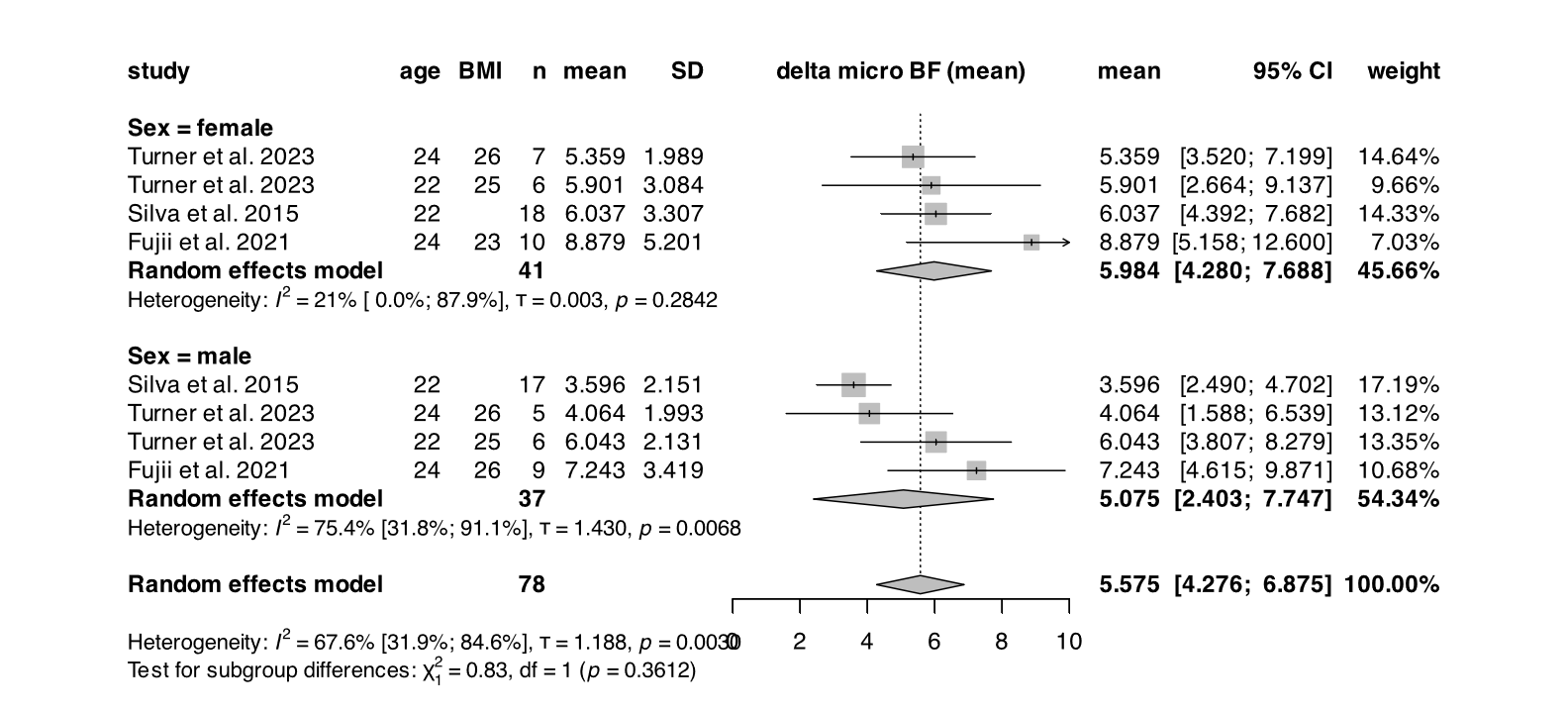
**

**Figure S21. Forest plot of the mean microΔBF stratified by sex.** Vertical lines represent the point estimate, horizontal lines represent the 95 % CI, and squares represent the weight for each study. Diamond represents the pooled mean difference in microΔBF (%). (BMI: body mass index, n: count, SD: standard deviation, microΔBF: relative change in blood flow in microcirculation (%), 95% CI: 95% confidence interval)

**
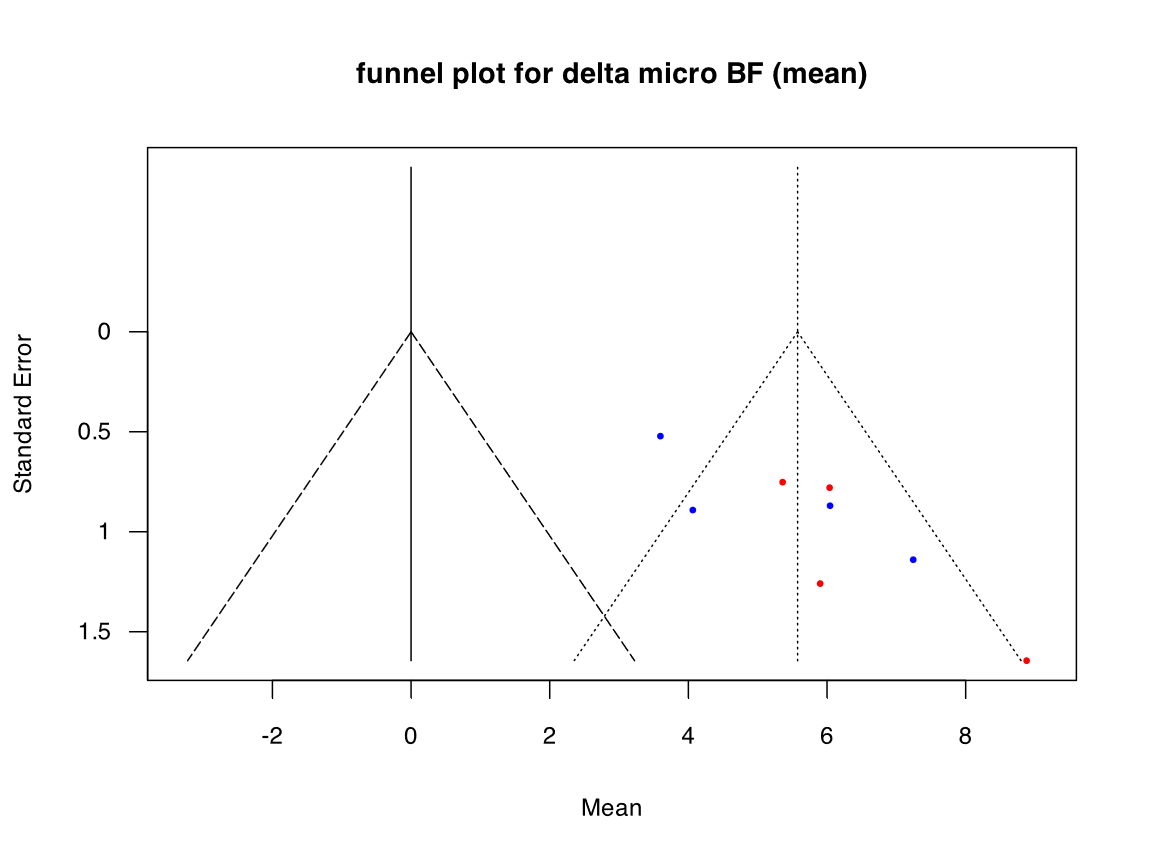
**

**Figure S22. Funnel plot for cutaneous mean microΔBF, stratified by sex (blue: male; red: female**). (microΔBF: relative change in blood flow in microcirculation (%))


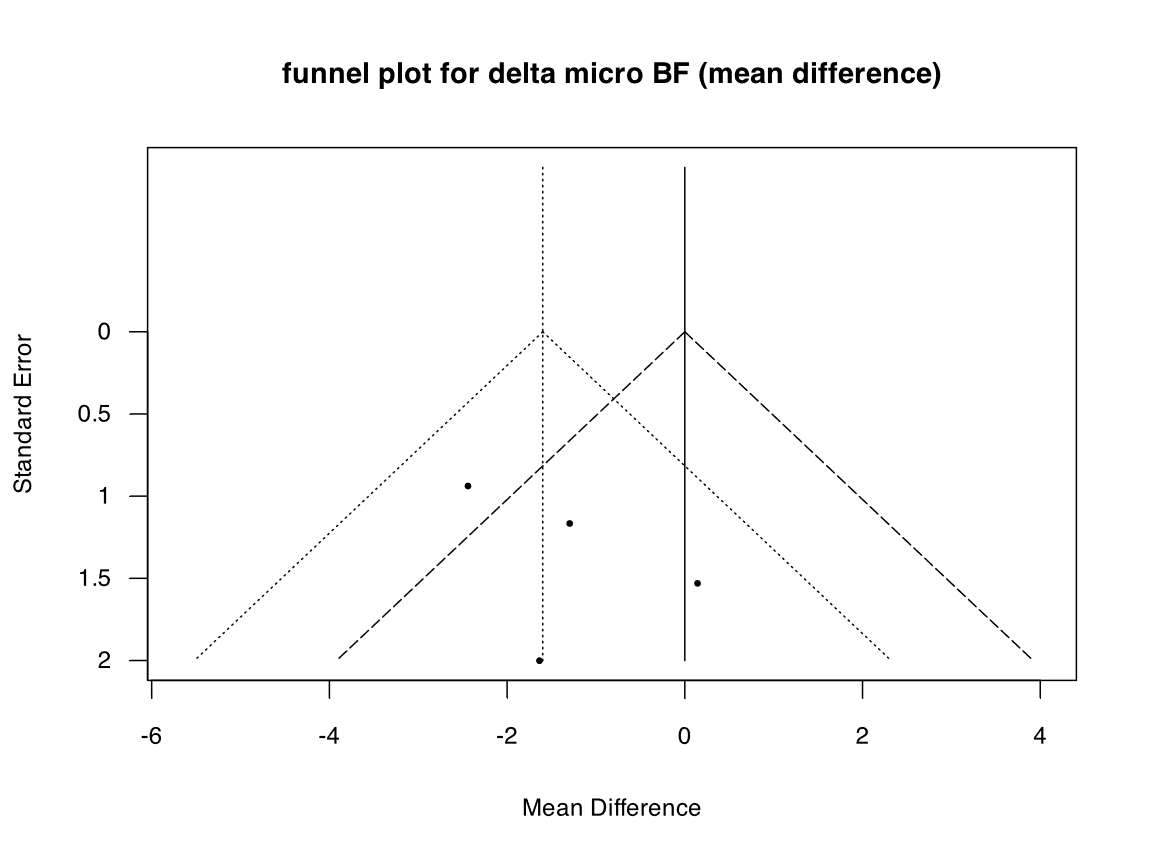


**Figure S23. Funnel plot for mean difference in microΔBF between sexes.** (microΔBF: relative change in blood flow in microcirculation (%))

| **Section and Topic** | **Item #** | **Checklist item** | **Reported (Yes/No)** |
| --- | --- | --- | --- |
| **TITLE** | | |  |
| Title | 1 | Identify the report as a systematic review. | Yes |
| **BACKGROUND** | | |  |
| Objectives | 2 | Provide an explicit statement of the main objective(s) or question(s) the review addresses. | Yes |
| **METHODS** | | |  |
| Eligibility criteria | 3 | Specify the inclusion and exclusion criteria for the review. | No-excl |
| Information sources | 4 | Specify the information sources (e.g. databases, registers) used to identify studies and the date when each was last searched. | No-date |
| Risk of bias | 5 | Specify the methods used to assess risk of bias in the included studies. | Yes |
| Synthesis of results | 6 | Specify the methods used to present and synthesise results. | No |
| **RESULTS** | | |  |
| Included studies | 7 | Give the total number of included studies and participants and summarise relevant characteristics of studies. | Yes |
| Synthesis of results | 8 | Present results for main outcomes, preferably indicating the number of included studies and participants for each. If meta-analysis was done, report the summary estimate and confidence/credible interval. If comparing groups, indicate the direction of the effect (i.e. which group is favoured). | Yes |
| **DISCUSSION** | | |  |
| Limitations of evidence | 9 | Provide a brief summary of the limitations of the evidence included in the review (e.g. study risk of bias, inconsistency and imprecision). | No |
| Interpretation | 10 | Provide a general interpretation of the results and important implications. | Yes |
| **OTHER** | | |  |
| Funding | 11 | Specify the primary source of funding for the review. | No |
| Registration | 12 | Provide the register name and registration number. | No |

**Table S1. PRISMA 2020 for Abstract Checklist.**

*From:*  Page MJ, McKenzie JE, Bossuyt PM, Boutron I, Hoffmann TC, Mulrow CD, et al. The PRISMA 2020 statement: an updated guideline for reporting systematic reviews. BMJ 2021;372:n71. doi: 10.1136/bmj.n71. This work is licensed under CC BY 4.0. To view a copy of this license, visit <https://creativecommons.org/licenses/by/4.0/>

**Table S2. PRISMA 2020 Checklist.**

| **Section and Topic** | **Item #** | **Checklist item** | **Location where item is reported** |
| --- | --- | --- | --- |
| **TITLE** | | |  |
| Title | 1 | Identify the report as a systematic review. | p1 |
| **ABSTRACT** | | |  |
| Abstract | 2 | See the PRISMA 2020 for Abstracts checklist. | Table S1 (S p 21) |
| **INTRODUCTION** | | |  |
| Rationale | 3 | Describe the rationale for the review in the context of existing knowledge. | p6 |
| Objectives | 4 | Provide an explicit statement of the objective(s) or question(s) the review addresses. | p6-7 |
| **METHODS** | | |  |
| Eligibility criteria | 5 | Specify the inclusion and exclusion criteria for the review and how studies were grouped for the syntheses. | p8 |
| Information sources | 6 | Specify all databases, registers, websites, organisations, reference lists and other sources searched or consulted to identify studies. Specify the date when each source was last searched or consulted. | p8 |
| Search strategy | 7 | Present the full search strategies for all databases, registers and websites, including any filters and limits used. | p8, Document S1 |
| Selection process | 8 | Specify the methods used to decide whether a study met the inclusion criteria of the review, including how many reviewers screened each record and each report retrieved, whether they worked independently, and if applicable, details of automation tools used in the process. | p8 |
| Data collection process | 9 | Specify the methods used to collect data from reports, including how many reviewers collected data from each report, whether they worked independently, any processes for obtaining or confirming data from study investigators, and if applicable, details of automation tools used in the process. | p8 |
| Data items | 10a | List and define all outcomes for which data were sought. Specify whether all results that were compatible with each outcome domain in each study were sought (e.g. for all measures, time points, analyses), and if not, the methods used to decide which results to collect. | p8 |
|  | 10b | List and define all other variables for which data were sought (e.g. participant and intervention characteristics, funding sources). Describe any assumptions made about any missing or unclear information. | p8 |
| Study risk of bias assessment | 11 | Specify the methods used to assess risk of bias in the included studies, including details of the tool(s) used, how many reviewers assessed each study and whether they worked independently, and if applicable, details of automation tools used in the process. | p8-9 |
| Effect measures | 12 | Specify for each outcome the effect measure(s) (e.g. risk ratio, mean difference) used in the synthesis or presentation of results. | p9 |
| Synthesis methods | 13a | Describe the processes used to decide which studies were eligible for each synthesis (e.g. tabulating the study intervention characteristics and comparing against the planned groups for each synthesis (item #5)). | p9 |
|  | 13b | Describe any methods required to prepare the data for presentation or synthesis, such as handling of missing summary statistics, or data conversions. | p9 |
|  | 13c | Describe any methods used to tabulate or visually display results of individual studies and syntheses. | p9 |
|  | 13d | Describe any methods used to synthesize results and provide a rationale for the choice(s). If meta-analysis was performed, describe the model(s), method(s) to identify the presence and extent of statistical heterogeneity, and software package(s) used. | p9 |
|  | 13e | Describe any methods used to explore possible causes of heterogeneity among study results (e.g. subgroup analysis, meta-regression). | p9 |
|  | 13f | Describe any sensitivity analyses conducted to assess robustness of the synthesized results. | p9 |
| Reporting bias assessment | 14 | Describe any methods used to assess risk of bias due to missing results in a synthesis (arising from reporting biases). | p9 |
| Certainty assessment | 15 | Describe any methods used to assess certainty (or confidence) in the body of evidence for an outcome. | p9 |
| **RESULTS** | | |  |
| Study selection | 16a | Describe the results of the search and selection process, from the number of records identified in the search to the number of studies included in the review, ideally using a flow diagram. | p10 |
|  | 16b | Cite studies that might appear to meet the inclusion criteria, but which were excluded, and explain why they were excluded. | Table S3 |
| Study characteristics | 17 | Cite each included study and present its characteristics. | Table S4-5 |
| Risk of bias in studies | 18 | Present assessments of risk of bias for each included study. | Table S6 |
| Results of individual studies | 19 | For all outcomes, present, for each study: (a) summary statistics for each group (where appropriate) and (b) an effect estimate and its precision (e.g. confidence/credible interval), ideally using structured tables or plots. | p10-11 |
| Results of syntheses | 20a | For each synthesis, briefly summarise the characteristics and risk of bias among contributing studies. | p10-11, Table S6 |
|  | 20b | Present results of all statistical syntheses conducted. If meta-analysis was done, present for each the summary estimate and its precision (e.g. confidence/credible interval) and measures of statistical heterogeneity. If comparing groups, describe the direction of the effect. | p10-11 |
|  | 20c | Present results of all investigations of possible causes of heterogeneity among study results. | p11 |
|  | 20d | Present results of all sensitivity analyses conducted to assess the robustness of the synthesized results. | p11 |
| Reporting biases | 21 | Present assessments of risk of bias due to missing results (arising from reporting biases) for each synthesis assessed. | p11, Figure S3-4, S7-8, S11-12, S15-16, S19-20, S22-23 |
| Certainty of evidence | 22 | Present assessments of certainty (or confidence) in the body of evidence for each outcome assessed. | p10-11 |
| **DISCUSSION** | | |  |
| Discussion | 23a | Provide a general interpretation of the results in the context of other evidence. | p12-14 |
|  | 23b | Discuss any limitations of the evidence included in the review. | p14-15 |
|  | 23c | Discuss any limitations of the review processes used. | p14-15 |
|  | 23d | Discuss implications of the results for practice, policy, and future research. | p15 |
| **OTHER INFORMATION** | | |  |
| Registration and protocol | 24a | Provide registration information for the review, including register name and registration number, or state that the review was not registered. | p8 |
|  | 24b | Indicate where the review protocol can be accessed, or state that a protocol was not prepared. | p8 |
|  | 24c | Describe and explain any amendments to information provided at registration or in the protocol. | - |
| Support | 25 | Describe sources of financial or non-financial support for the review, and the role of the funders or sponsors in the review. | p2 |
| Competing interests | 26 | Declare any competing interests of review authors. | p2 |
| Availability of data, code and other materials | 27 | Report which of the following are publicly available and where they can be found: template data collection forms; data extracted from included studies; data used for all analyses; analytic code; any other materials used in the review. | p2 |

*From:*  Page MJ, McKenzie JE, Bossuyt PM, Boutron I, Hoffmann TC, Mulrow CD, et al. The PRISMA 2020 statement: an updated guideline for reporting systematic reviews. BMJ 2021;372:n71. doi: 10.1136/bmj.n71. This work is licensed under CC BY 4.0. To view a copy of this license, visit <https://creativecommons.org/licenses/by/4.0/>

**Table S3. List of articles that were left out from the meta-analysis.**

| **Author** | **Reason for exclusion from meta-analysis** |
| --- | --- |
| Akhundova, J. 2021 ^1^ | Undeclared smoking status |
| Herrington, D. M. 2001 ^2^ | Undeclared smoking status |
| Karthik, S. 2019 ^3^ | Undeclared smoking status |
| Morales-Acuna, F. 2019 ^4^ | Undeclared smoking status |
| Credeur, D. P. 2010 ^5^ | Undeclared smoking status |
| Dengel, D. R. 2011 ^6^ | Undeclared smoking status |
| Levenson, J. 2001 ^7^ | Undeclared smoking status |
| Lalande, S. 2021 ^8^ | Undeclared smoking status |
| Yufu, K. 2009 ^9^ | Undeclared smoking status |
| Blum, A. 2019 ^10^ | Undeclared smoking status |
| Funada, J. 2010 ^11^ | Undeclared smoking status |
| Johns, J. A. 2020 ^12^ | Undeclared smoking status |
| Chumaeva, N. 2010 ^13^ | Undeclared smoking status |
| Rathod, K. S. 2017 ^14^ | Undeclared smoking status |
| Rudolph, T. K. 2007 ^15^ | Undeclared smoking status |
| Shenouda, N. 2018 ^16^ | Undeclared smoking status |
| Habib, K. 2022 ^17^ | Undeclared smoking status |
| Järvela, L. S. 2013 ^18^ | Undeclared smoking status |
| Ratcliffe, B. 2017 ^19^ | Undeclared smoking status |
| Weggen, J. B. 2021 ^20^ | Undeclared smoking status |
| Violanti, J. M. 2006 ^21^ | Undeclared smoking status |
| de Jongh, R. T. 2007 ^22^ | Undeclared smoking status |
| Gryglewska, B. 2009 ^23^ | Undeclared smoking status |
| Zócalo, Y. 2021 ^24^ | wide age range |
| Königstein, K. 2022 ^25^ | wide age range |
| Vogel, R. A. 2000 ^26^ | wide age range |
| Plavnik, F. L. 2007 ^27^ | wide age range |
| Jensen-Urstad, K. 1997 ^28^ | wide age range |
| Hamburg, N. M. 2011 ^29^ | wide age range |
| Ardigo, D. 2006 ^30^ | wide age range |
| Thomas, G. N. 2008 ^31^ | wide age range |
| Ardigo, D. 2007 ^32^ | wide age range |
| Königstein, K. 2021 ^33^ | non- poolable outcome measure |
| Regensteiner, J. G. 2015 ^34^ | non- poolable outcome measure |
| Province, V.M. 2022 ^35^ | non- poolable outcome measure |
| Renata, S. 2011 ^36^ | non- poolable outcome measure |
| Yvonne-Tee, G. B. 2008 ^37^ | non- poolable outcome measure |
| Stupin, A. 2019 ^38^ | non- poolable outcome measure |
| Rodrigues, L. M. 2001 ^39^ | non- poolable outcome measure |
| Migdal, K. U. 2020 ^40^ | missing information |
| Yang, P. T. 2014 ^41^ | missing information |

**Table S4.** **Main characteristics of the studies of the brachial artery, included in the systematic review and meta-analysis.** Data are represented as mean ± standard deviation. (N*: count, BMI†: body mass index, FMD‡: flow-mediated dilation, BAD§: baseline artery diameter, ΔBF||: relative change in blood flow on the brachial artery, WSR#: wall shear rate, TTP**: time-to-peak)

| **Study characteristics** | | **Demography** | | | | | | | |  |
| --- | --- | --- | --- | --- | --- | --- | --- | --- | --- | --- |
| **Author** | **Study design** | **Occlusion site** | **Country** | **Gender** | **N*** | **Age** | **Age SD** | **BMI†** | **BMI SD** | **Outcome** |
| Celermajer, D. S. et al. 1994 ^42^ | Cross-sectional study | forearm | USA | male | 12 | 25.8 | 3.2 | NA | NA | FMD‡ |
|  |  |  |  | female | 20 | 27.1 | 1.8 | NA | NA |  |
|  |  |  |  | male | 24 | 35 | 2.7 | NA | NA |  |
|  |  |  |  | female | 16 | 34.8 | 3.8 | NA | NA |  |
| Perregaux, D. et al. 2000 ^43^ | Cross-sectional study | forearm | USA | male | 12 | 29.5 | 5.9 | 27.0 | 3.8 | FMD, BAD§, ΔBF\|\| |
|  |  |  |  | female | 12 | 29.5 | 5.9 | 27.0 | 3.8 |  |
| Hamburg, N. M. et al. 2004 ^44^ | Cross-sectional study | upper arm | USA | male | 13 | 33 | 3.6 | 24.0 | 2.5 | FMD, BAD |
|  |  |  |  | female | 15 | 31 | 3.9 | 22.0 | 2.3 |  |
| Hurks, R. et al. 2009 ^45^ | Cross-sectional study | forearm | Belgium | male | 16 | 23.1 | 2.8 | 21.1 | 1.7 | FMD |
|  |  |  |  | female | 20 | 23.1 | 2.8 | 21.1 | 1.7 |  |
| Lin, M. et al. 2014 ^46^ | Cross-sectional study | forearm | Taiwan | male | 21 | 30 | 7.0 | 22.3 | 2.3 | FMD, ΔBF |
|  |  |  |  | female | 21 | 30 | 7.0 | 20.3 | 2.3 |  |
| Perregaux, D. et al. 1999 ^47^ | Cross-sectional study | forearm | USA | male | 14 | 26 | 5.2 | 26.0 | 7.5 | FMD, BAD, ΔBF |
|  |  |  |  | female | 14 | 27 | 3.0 | 22.0 | 3.4 |  |
| Corretti, M. C. et al. 1995 ^48^ | Cross-sectional study | upper arm | USA | male | 10 | 33 | 3.0 | NA | NA | FMD, BAD, ΔBF |
|  |  |  |  | female | 10 | 32 | 4.0 | NA | NA |  |
| Lan, W. R. et al. 2011 ^49^ | Prospective clinical trial, cross-sectional data were used | forearm | Taiwan | male | 16 | 30.4 | 2.0 | 21.7 | 1.9 | FMD |
|  |  |  |  | female | 7 | 30.4 | 2.0 | 21.7 | 1.9 |  |
| Paradossi, U. et al. 2004 ^50^ | Cross-sectional study | upper arm | Italy | male | 46 | 30.8 | 5.4 | NA | NA | FMD, BAD |
|  |  |  |  | female | 72 | 29.7 | 5.1 | NA | NA |  |
| Tremblay, J. C. et al. 2019 ^51^ | Prospective clinical trial, cross-sectional data were used | forearm | Canada | male | 16 | 25 | 3.0 | 24.1 | 2.5 | FMD, BAD, WSR#, TTP** |
|  |  |  |  | female | 16 | 21 | 2.0 | 23.0 | 2.7 |  |
| Tremblay, J. C. et al. 2019 ^52^ | Cross-sectional study | forearm | Canada | male | 12 | 23 | 2.0 | 24.9 | 2.0 | FMD, BAD, WSR, TTP |
|  |  |  |  | female | 12 | 20 | 2.0 | 24.2 | 3.0 |  |
| Nair, B. M. et al. 2004 ^53^ | Cross-sectional study | forearm | India | male | 12 | 32 | 7.0 | 26.0 | 3.6 | FMD |
|  |  |  |  | female | 8 | 32 | 7.0 | 26.0 | 3.6 |  |
| Mashayekhi, G. et al. 2015 ^54^ | Cross-sectional study | upper arm | Iran | male | 10 | 27.5 | 3.0 | NA | NA | FMD |
|  |  |  |  | female | 7 | 27.5 | 3.0 | NA | NA |  |
| Parker, B. A. et al. 2011 ^55^ | Prospective clinical trial, cross-sectional data were used | forearm | Canada | male | 8 | 29 | 4.0 | 25.4 | 3.0 | FMD |
|  |  |  |  | female | 8 | 28 | 5.0 | 24.7 | 2.5 |  |
| Hanson, B. E. et al. 2022 ^56^ | Prospective clinical trial, cross-sectional data were used | forearm | USA | male | 8 | 23 | 5.0 | 24.5 | 4.0 | FMD |
|  |  |  |  | female | 4 | 23 | 5.0 | 24.5 | 4.0 |  |
| Hwang, I. C. et al. 2012 ^57^ | Prospective clinical trial, cross-sectional data were used | forearm | Korea | male | 35 | 22.26 | 2.3 | 22.3 | 2.1 | FMD, BAD |
|  |  |  |  | female | 39 | 23.21 | 3.0 | 20.5 | 1.8 |  |
| Jiang, X. B. et al. 2013 ^58^ | Cross-sectional study | forearm | China | male | 18 | 30.37 | 8.5 | 21.6 | 2.5 | FMD |
|  |  |  |  | female | 42 | 30.37 | 8.5 | 21.6 | 2.5 |  |
| Lindkvist, M. et al. 2019 ^59^ | Cross-sectional study | forearm | Sweden | male | 16 | 22 | 2.0 | 23.0 | 2.0 | FMD |
|  |  |  |  | female | 27 | 22 | 2.0 | 23.0 | 3.0 |  |
| Petrovic, D. J. et al. 2023 ^60^ | Cross-sectional study | upper arm | Serbia | male | 5 | 34.7 | 3.7 | NA | NA | FMD, BAD |
|  |  |  |  | female | 6 | 30.3 | 3.3 | NA | NA |  |
| Hanson, B. E. et al. 2023 ^61^ | Prospective clinical trial, cross-sectional data were used | forearm | USA | male | 9 | 22 | 4.0 | 23.3 | 3.7 | FMD |
|  |  |  |  | female | 4 | 22 | 4.0 | 23.3 | 3.7 |  |
| Safdar, B. et al. 2016 ^62^ | Prospective clinical trial, cross-sectional data were used | forearm | USA | male | 10 | 31.48 | 8.3 | 25.0 | 9.5 | FMD, BAD |
|  |  |  |  | female | 11 | 29.1 | 7.5 | 24.0 | 9.9 |  |
| Barton, M. et al. 2011 ^63^ | Cross-sectional study | forearm | UK | male | 12 | 27 | 7.2 | 22.6 | 3.8 | FMD, BAD |
|  |  |  |  | female | 18 | 27 | 7.2 | 22.6 | 3.8 |  |
| Hashimoto, M. et al. 1995 ^64^ | Prospective clinical trial, cross-sectional data were used | forearm | Japan | male | 17 | 24.8 | 2.5 | 22.4 | 5.8 | FMD, BAD, ΔBF |
|  |  |  |  | female | 17 | 25.1 | 3.3 | 20.5 | 2.1 |  |
| Uehata, A. et al. 1997 ^65^ | Cross-sectional study | upper arm | USA | male | 10 | 31.7 | 2.7 | NA | NA | FMD, BAD, |
|  |  |  |  | female | 10 | 31.3 | 3.0 | NA | NA |  |
| Celermajer, D. S. et al. 1996 ^66^ | Cross-sectional study | forearm | USA | male | 13 | 22 | 4.0 | NA | NA | FMD |
|  |  |  |  | female | 13 | 22 | 4.0 | NA | NA |  |
| Campia, U. et al. 2002 ^67^ | Cross-sectional study | forearm | USA | male | 23 | 37 | 8.0 | 28.1 | 4.3 | FMD, BAD, ΔBF |
|  |  |  |  | female | 23 | 38 | 9.0 | 28.1 | 4.3 |  |
|  |  |  |  | male | 23 | 38 | 11.0 | 24.4 | 2.9 |  |
|  |  |  |  | female | 23 | 36 | 9.0 | 24.4 | 2.9 |  |
| Nishiyama, S. K. et al. 2008 ^68^ | Cross-sectional study | upper arm | USA | male | 15 | 26 | 7.7 | NA | NA | FMD, BAD, WSR |
|  |  |  |  | female | 15 | 23 | 3.9 | NA | NA |  |
| Ratchford, S. M. et al. 2021 ^69^ | Prospective clinical trial, cross-sectional data were used | forearm | USA | male | 5 | 22.6 | 1.1 | 20.8 | 1.7 | FMD, BAD, WSR, TTP |
|  |  |  |  | female | 15 | 23.2 | 1.3 | 23.1 | 2.1 |  |
| Paradossi, U. et al. 2012 ^70^ | Cross-sectional study | upper arm | Italy | male | 16 | 30.8 | 6.2 | 23.5 | 2.0 | FMD, BAD |
|  |  |  |  | female | 22 | 30.8 | 6.2 | 21.6 | 3.1 |  |
|  |  |  |  | male | 33 | 30.5 | 5.8 | 23.5 | 2.3 |  |
|  |  |  |  | female | 29 | 30.5 | 5.8 | 21.4 | 2.4 |  |
| Akins, J. D. et al. 2022 ^71^ | Cross-sectional study | forearm | USA | male | 15 | 23 | 3.0 | 23.7 | 3.2 | FMD, BAD, ΔBF, WSR, TTP |
|  |  |  |  | female | 11 | 22 | 4.0 | 23.8 | 4.2 |  |
| Plotnik, G. et al. 1997 ^72^ | Prospective clinical trial, cross-sectional data were used | upper arm | USA | male | 7 | 37.3 | 9.1 | 23.0 | 4.0 | FMD, BAD |
|  |  |  |  | female | 13 | 37.3 | 9.1 | 23.0 | 4.0 |  |
| Germain, A. et al. 2004 ^73^ | Cross-sectional study | forearm | Chile | male | 22 | 22.4 | 12.2 | 23.1 | 9.4 | FMD |
|  |  |  |  | female | 24 | 33.3 | 20.1 | 22.8 | 14.7 |  |
| Shenouda, N. et al. 2018 ^74^ | Prospective clinical trial, cross-sectional data were used | forearm | Canada | male | 8 | 22 | 4.0 | 27 | 6.0 | FMD, BAD, TTP |
|  |  |  |  | female | 8 | 21 | 3.0 | 24 | 6.0 |  |
| Soares, C.M. et al. 2010 ^75^ | Cross-sectional study | forearm | Brazil | male | 18 | NA | NA | NA | NA | FMD |
|  |  |  |  | female | 24 | NA | NA | NA | NA |  |
| Nakamura, A. et al. 2019 ^76^ | Cross-sectional study | forearm | Japan | male | 17 | 26 | 2.0 | 22 | 2.0 | FMD, BAD |
|  |  |  |  | female | 16 | 27 | 2.0 | 19 | 1.0 |  |
| Black, M.A. et al. 2009 ^77^ | Prospective clinical trial, cross-sectional data were used | forearm | UK | male | 9 | 26 | 3.3 | 23 | 3.0 | FMD, BAD WSR |
|  |  |  |  | female | 9 | 26 | 3.6 | 23 | 3.0 |  |
| Skow, R. et al. 2022 ^78^ | Cross-sectional study | forearm | USA | male | 6 | 26 | 4.0 | 25.7 | 4.1 | FMD |
|  |  |  |  | female | 7 | 26 | 4.0 | 25.7 | 4.1 |  |
| Morishima, T. et al. 2020 ^79^ | Prospective clinical trial, cross-sectional data were used | forearm | Japan | male | 16 | 19.6 | 1.4 | 23.3 | 4.3 | FMD, BAD WSR, TTP |
|  |  |  |  | female | 13 | 19.5 | 0.7 | 20.9 | 2.3 |  |
| Harris, R.A. et al. 2012 ^80^ | Prospective clinical trial, cross-sectional data were used | forearm | USA | male | 10 | 23 | 2.8 | 23 | 3.2 | FMD, BAD, WSR, TTP |
|  |  |  |  | female | 15 | 20 | 1.9 | 24 | 3.9 |  |
| Holder, S.M. et al. 2018 ^81^ | Prospective clinical trial, cross-sectional data were used | forearm | UK | male | 389 | 25 | 5.0 | 23.6 | 2.8 | FMD, BAD, WSR, TTP |
|  |  |  |  | female | 144 | 27 | 6.0 | 24.6 | 5.2 |  |
| Lennon-Edwards, S. et al. 2014 ^82^ | Prospective clinical trial, cross-sectional data were used | forearm | USA | male | 16 | 29 | 8.0 | 23.8 | 2.8 | FMD, BAD, WSR |
|  |  |  |  | female | 14 | 31 | 7.5 | 24.5 | 2.6 |  |
| Gentilin, A. et al. 2022 ^83^ | Prospective clinical trial, cross-sectional data were used | forearm | Italy | male | 12 | 25.6 | 3.7 | NA | NA | FMD, BAD, ΔBF, WSR |
|  |  |  |  | female | 13 | 23.8 | 2.6 | NA | NA |  |
| Badrov, M.B. et al 2015 ^84^ | Prospective clinical trial, cross-sectional data were used | forearm | Canada | male | 9 | 21 | 2.0 | 24 | 2.0 | FMD, BAD, WSR, TTP |
|  |  |  |  | female | 11 | 23 | 4.0 | 21 | 2.0 |  |
| Arbeitman, C.R. et al. 2016 ^85^ | Cross-sectional study | forearm | Argentina | male | 5 | 24.4 | 5.6 | 22.4 | 1.5 | FMD |
|  |  |  |  | female | 5 | 28 | 12.2 | 25 | 1.8 |  |
| King, T.J. et al. 2023 ^86^ | Prospective clinical trial, cross-sectional data were used | forearm | Canada | male | 19 | 23 | 5.0 | 23.4 | 3.1 | FMD, BAD |
|  |  |  |  | female | 10 | 23 | 5.0 | 23.4 | 3.1 |  |
| Tomiyama, H. et al. 2008 ^87^ | Cross-sectional study | forearm | Japan | male | 177 | NA | NA | 23.7 | 3.1 | FMD, BAD |
|  |  |  |  | female | 113 | NA | NA | 21.1 | 2.7 |  |
| Kim, W. et al. 2000 ^88^ | Cross-sectional study | forearm | South-Korea | male | 8 | 28 | 5.0 | 22.8 | 4.0 | FMD, BAD, ΔBF |
|  |  |  |  | female | 9 | 27 | 4.0 | 19.8 | 2.6 |  |
| Smiljanec, K. et al. 2020 ^89^ | randomized controlled crossover trial, cross-sectional data were used | forearm | USA | male | 20 | 26 | 4.5 | 24.3 | 2.2 | FMD, BAD, WSR |
|  |  |  |  | female | 21 | 22 | 4.6 | 22.5 | 2.3 |  |

**Table S5. The main characteristics of skin studies included in the systematic review and meta-analysis.** Data are represented as mean ± standard deviation. (N*: count, BMI†: body mass index, microΔBF‡: relative change in blood flow in microcirculation)

| **Study characteristics** | | | | **Demography** | | | | | |  |
| --- | --- | --- | --- | --- | --- | --- | --- | --- | --- | --- |
| **Author** | **Study design** | **Measurement site** | **Country** | **Sex** | **N*** | **Age** | **Age SD** | **BMI†** | **BMI SD** | **Outcome** |
| Turner, C. G. et al. 2023 ^90^ | Cross-sectional study | forearm skin | USA | male | 6 | 22 | 3 | 25.2 | 2.7 | microΔBF‡ |
|  |  |  |  | female | 6 | 22 | 3 | 25.2 | 2.7 |  |
|  |  |  |  | male | 5 | 24 | 2 | 25.5 | 2.4 |  |
|  |  |  |  | female | 7 | 24 | 2 | 25.5 | 2.4 |  |
| Fujii, N. et al. 2021 ^91^ | Cross-sectional study | forearm skin | Canada | male | 9 | 24 | 4 | 25.7 | 1.81 | microΔBF |
|  |  |  |  | female | 10 | 24 | 4 | 23.1 | 1.89 |  |
| Silva, H. et al. 2015 ^92^ | Cross-sectional study | toe skin | Portugal | male | 17 | 22,1 | 3.7 | NA | NA | microΔBF |
|  |  |  |  | female | 18 | 22,1 | 3.7 | NA | NA |  |

**Table S6. Tabular display of Risk of bias assessment with Checklist For Analytical Cross Sectional Studies, Critical Appraisal tools for use in JBI Systematic Reviews.**

| **Author** | **Year** | **1.** | **2.** | **3.** | **4.** | **5.** | **6.** | **7.** | **8.** | **Overall Score** |
| --- | --- | --- | --- | --- | --- | --- | --- | --- | --- | --- |
| Celermajer, D. S et al. | 1994 | N | N | Y | Y | N | NA | Y | Y | 4 |
| Perregaux, D. et al. | 2000 | Y | Y | Y | Y | Y | Y | Y | Y | 8 |
| Hamburg, N. M. et al. | 2004 | N | Y | Y | Y | N | NA | Y | Y | 5 |
| Hurks, R. et al. | 2009 | Y | Y | Y | Y | N | NA | Y | Y | 6 |
| Lin, M. et al. | 2014 | Y | Y | Y | Y | N | NA | Y | Y | 6 |
| Perregaux, D. et al. | 1999 | Y | Y | Y | Y | N | NA | Y | Y | 6 |
| Corretti, M. C. et al. | 1995 | N | N | Y | Y | N | Y | Y | Y | 5 |
| Lan, W. R. et al. | 2011 | Y | Y | Y | Y | N | NA | Y | Y | 6 |
| Paradossi, U. et al. | 2004 | N | N | Y | Y | N | NA | Y | Y | 4 |
| Tremblay, J. C. et al. | 2019 | N | Y | Y | Y | Y | NA | Y | Y | 6 |
| Tremblay, J. C. et al. | 2019 | N | Y | Y | Y | Y | NA | Y | Y | 6 |
| Nair, B. M. et al. | 2004 | Y | Y | Y | Y | N | NA | Y | Y | 6 |
| Mashayekhi, G. et al. | 2015 | Y | Y | Y | Y | N | NA | Y | Y | 6 |
| Parker, B. A. et al. | 2011 | Y | Y | Y | Y | Y | Y | Y | Y | 8 |
| Hanson, B. E. et al. | 2022 | N | Y | Y | Y | Y | Y | Y | Y | 7 |
| Hwang, I. C. et al. | 2012 | Y | Y | Y | Y | N | NA | Y | Y | 6 |
| Jiang, X. B. et al. | 2014 | Y | Y | Y | Y | N | NA | Y | Y | 6 |
| Lindkvist, M. et al. | 2019 | Y | Y | Y | Y | N | NA | Y | Y | 6 |
| Petrovic, D. J. et al. | 2023 | Y | Y | Y | Y | N | NA | Y | Y | 6 |
| Hanson, B. E. et al. | 2023 | Y | Y | Y | Y | Y | Y | Y | Y | 8 |
| Safdar, B. et al. | 2016 | Y | Y | Y | Y | Y | Y | Y | Y | 8 |
| Barton, M. et al. | 2011 | Y | Y | Y | Y | N | NA | Y | Y | 6 |
| Hashimoto, M. et al. | 1995 | Y | Y | Y | Y | Y | Y | Y | Y | 8 |
| Uehata, A. et al. | 1997 | N | N | Y | Y | N | NA | Y | Y | 4 |
| Celermajer, D. S. et al. | 1996 | N | N | Y | Y | N | NA | Y | Y | 4 |
| Campia, U. et al. | 2002 | Y | Y | Y | Y | N | NA | Y | Y | 6 |
| Nishiyama, S. K. et al. | 2008 | Y | Y | Y | Y | Y | Y | Y | Y | 8 |
| Ratchford, S. M. et al. | 2021 | Y | Y | Y | Y | Y | N | Y | Y | 7 |
| Paradossi, U. et al. | 2012 | Y | Y | Y | Y | N | NA | Y | Y | 6 |
| Akins, J. D. et al. | 2022 | Y | Y | Y | Y | N | NA | Y | Y | 6 |
| Plotnik, G. et al. | 1997 | Y | Y | Y | Y | Y | Y | Y | Y | 8 |
| Germain, A. et al. | 2004 | Y | Y | Y | Y | Y | Y | Y | Y | 8 |
| Shenouda, N. et al. | 2018 | Y | Y | Y | Y | Y | Y | Y | Y | 8 |
| Soares, C.M. et al. | 2010 | N | N | Y | Y | N | NA | Y | Y | 4 |
| Nakamura, A. et al. | 2019 | N | Y | Y | Y | Y | Y | Y | Y | 7 |
| Black, M.A. et al. | 2009 | Y | Y | Y | Y | Y | Y | Y | Y | 8 |
| Skow, R. et al. | 2022 | N | U | Y | Y | N | NA | Y | Y | 4 |
| Morishima, T. et al. | 2020 | Y | Y | Y | Y | N | NA | Y | Y | 6 |
| Harris, R. A. et al. | 2012 | Y | Y | Y | Y | Y | Y | Y | Y | 8 |
| Holder, S.M. et al. | 2018 | U | Y | Y | Y | Y | Y | Y | Y | 7 |
| Lennon-Edwards, S. et al. | 2014 | Y | Y | Y | Y | N | NA | Y | Y | 6 |
| Gentilin, A. et al. | 2022 | Y | Y | Y | Y | Y | Y | Y | Y | 8 |
| Badrov, M. A. et al. | 2015 | N | Y | Y | Y | Y | Y | Y | Y | 7 |
| Arbeitman, C. R. et al. | 2016 | Y | Y | Y | Y | N | NA | Y | Y | 6 |
| King, T.J. et al. | 2023 | Y | Y | Y | Y | Y | Y | Y | Y | 8 |
| Tomiyama, H. et al. | 2008 | Y | Y | Y | Y | Y | Y | Y | Y | 8 |
| Kim, W. et al. | 2000 | Y | Y | Y | Y | N | NA | Y | Y | 6 |
| Smiljanec, K. et al. | 2020 | N | Y | Y | Y | N | NA | Y | Y | 5 |
| Turner, C. G. et al. | 2023 | N | Y | Y | Y | N | NA | Y | Y | 7 |
| Fujii, N. et al. | 2021 | N | Y | Y | Y | Y | Y | Y | Y | 7 |
| Silva, H. et al. | 2015 | N | N | Y | Y | N | NA | Y | Y | 4 |

References

1. Akhundova J, Kaya CT, Gerede Uludağ DM. Acute effects of consumption of low-caffeine energy drinks on endothelial functions in healthy volunteers. *Anatol J Cardiol*. 2021;25:678-683

2. Herrington DM, Fan L, Drum M, Riley WA, Pusser BE, Crouse JR, et al. Brachial flow-mediated vasodilator responses in population-based research: Methods, reproducibility and effects of age, gender and baseline diameter. *J Cardiovasc Risk*. 2001;8:319-328

3. Karthik S, Vipin VP, Kapoor A, Tripathi A, Shukla M, Dabadghao P. Cardiovascular disease risk in the siblings of women with polycystic ovary syndrome. *Hum Reprod*. 2019;34:1559-1566

4. Morales-Acuna F, Ochoa L, Valencia C, Gurovich AN. Characterization of blood flow patterns and endothelial shear stress during flow-mediated dilation. *Clin Physiol Funct Imaging*. 2019;39:240-245

5. Credeur DP, Hollis BC, Welsch MA. Effects of handgrip training with venous restriction on brachial artery vasodilation. *Med Sci Sports Exerc*. 2010;42:1296-1302

6. Dengel DR, Jacobs DR, Steinberger J, Moran AM, Sinaiko AR. Gender differences in vascular function and insulin sensitivity in young adults. *Clin Sci (Lond)*. 2011;120:153-160

7. Levenson J, Pessana F, Gariepy J, Armentano R, Simon A. Gender differences in wall shear-mediated brachial artery vasoconstriction and vasodilation. *J Am Coll Cardiol*. 2001;38:1668-1674

8. Lalande S, Hemingway HW, Jarrard CP, Moore AM, Olivencia-Yurvati AH, Richey RE, et al. Influence of ischemia-reperfusion injury on endothelial function in men and women with similar serum estradiol concentrations. *Am J Physiol Regul Integr Comp Physiol*. 2021;321:R273-r278

9. Yufu K, Takahashi N, Okada N, Shinohara T, Hara M, Saikawa T, et al. Influence of systolic blood pressure and cigarette smoking on endothelial function in young healthy people. *Circ J*. 2009;73:174-178

10. Blum A, Pastukh N, Sirchan R, Blum N, Chernikoff L, Vaispapir V. Inhibition of endothelial progenitor cells inhibition in the first 24 hours of an acute ischemic cerebrovascular event. *Isr Med Assoc J*. 2019;21:71-76

11. Funada J, Dennis AL, Roberts R, Karpe F, Frayn KN. Regulation of subcutaneous adipose tissue blood flow is related to measures of vascular and autonomic function. *Clin Sci (Lond)*. 2010;119:313-322

12. Johns JA, O'Brien MW, Bungay A, Kimmerly DS. Sex and light physical activity impact popliteal, but not brachial artery flow-mediated dilation in physically active young adults. *Appl Physiol Nutr Metab*. 2020;45:1387-1395

13. Chumaeva N, Hintsanen M, Juonala M, Raitakari OT, Keltikangas-Järvinen L. Sex differences in the combined effect of chronic stress with impaired vascular endothelium functioning and the development of early atherosclerosis: The cardiovascular risk in young finns study. *BMC Cardiovasc.* 2010;10:34

14. Rathod KS, Kapil V, Velmurugan S, Khambata RS, Siddique U, Khan S, et al. Accelerated resolution of inflammation underlies sex differences in inflammatory responses in humans. *J Clin Invest*. 2017;127:169-182

15. Rudolph TK, Ruempler K, Schwedhelm E, Tan-Andresen J, Riederer U, Böger RH, et al. Acute effects of various fast-food meals on vascular function and cardiovascular disease risk markers: The hamburg burger trial. *Am J Clin Nutr*. 2007;86:334-340

16. Shenouda N, Priest SE, Rizzuto VI, MacDonald MJ. Brachial artery endothelial function is stable across a menstrual and oral contraceptive pill cycle but lower in premenopausal women than in age-matched men. *Am J Physiol Heart Circ Physiol*. 2018;315:H366-h374

17. Habib K, Fallah B, Edgell H. Effect of upright posture on endothelial function in women and men. *Front Physiol*. 2022;13:846229

18. Järvelä LS, Niinikoski H, Heinonen OJ, Lähteenmäki PM, Arola M, Kemppainen J. Endothelial function in long-term survivors of childhood acute lymphoblastic leukemia: Effects of a home-based exercise program. *Pediatr Blood Cancer*. 2013;60:1546-1551

19. Ratcliffe B, Pawlak R, Morales F, Harrison C, Gurovich AN. Internal validation of an automated system for brachial and femoral flow mediated dilation. *Clin Hypertens*. 2017;23:17

20. Weggen JB, Darling AM, Autler AS, Hogwood AC, Decker KP, Imthurn B, et al. Impact of acute antioxidant supplementation on vascular function and autonomic nervous system modulation in young adults with ptsd. *Am J Physiol Regul Integr Comp Physiol*. 2021;321:R49-r61

21. Violanti JM, Burchfiel CM, Miller DB, Andrew ME, Dorn J, Wactawski-Wende J, et al. The buffalo cardio-metabolic occupational police stress (bcops) pilot study: Methods and participant characteristics. *Ann Epidemiol*. 2006;16:148-156

22. de Jongh RT, Ijzerman RG, Serné EH, van Weissenbruch MM, Voordouw JJ, Delemarre-van de Waal HA, et al. Urinary cortisol is inversely associated with capillary recruitment in women: A potential explanation for the cortisol-blood pressure relationship. *Clin Sci (Lond)*. 2007;113:83-91

23. Gryglewska B, Nȩcki M, Cwynar M, Baron T, Grodzicki T. Effect of gender on skin forearm microcirculation among middle age subjects with newly diagnosed hypertension. *Nadcisnienie Tetnicze*. 2009;13:376-387

24. Zócalo Y, Bia D. Age- and sex-related profiles for macro, macro/micro and microvascular reactivity indexes: Association between indexes and normative data from 2609 healthy subjects (3-85 years). *PLoS One*. 2021;16:e0254869

25. Königstein K, Wagner J, Infanger D, Knaier R, Nève G, Klenk C, et al. Cardiorespiratory fitness and endothelial function in aging healthy subjects and patients with cardiovascular disease. *Front Cardiovasc Med*. 2022;9:870847

26. Vogel RA, Corretti MC, Plotnick GD. A comparison of brachial artery flow-mediated vasodilation using upper and lower arm arterial occlusion in subjects with and without coronary risk factors. *Clin Cardiol*. 2000;23:571-575

27. Plavnik FL, Ajzen SA, Christofalo DM, Barbosa CS, Kohlmann O, Jr. Endothelial function in normotensive and high-normal hypertensive subjects. *J Hum Hypertens*. 2007;21:467-472

28. Jensen-Urstad K, Rosfors S. A methodological study of arterial wall function using ultrasound technique. *Clin Physiol*. 1997;17:557-567

29. Hamburg NM, Palmisano J, Larson MG, Sullivan LM, Lehman BT, Vasan RS, et al. Relation of brachial and digital measures of vascular function in the community: The framingham heart study. *Hypertension*. 2011;57:390-396

30. Ardigo D, Franzini L, Valtuena S, Monti LD, Reaven GM, Zavaroni I. Relation of plasma insulin levels to forearm flow-mediated dilatation in healthy volunteers. *Am J Cardiol*. 2006;97:1250-1254

31. Thomas GN, Chook P, Yip TW, Kwong SK, Chan TY, Qiao M, et al. Smoking without exception adversely affects vascular structure and function in apparently healthy chinese: Implications in global atherosclerosis prevention. *Int J Cardiol*. 2008;128:172-177

32. Ardigo D, Stüehlinger M, Franzini L, Valtueña S, Piatti PM, Pachinger O, et al. Adma is independently related to flow-mediated vasodilation in subjects at low cardiovascular risk. *Eur J Clin Invest*. 2007;37:263-269

33. Königstein K, Wagner J, Frei M, Knaier R, Klenk C, Carrard J, et al. Endothelial function of healthy adults from 20 to 91 years of age: Prediction of cardiovascular risk by vasoactive range. *J Hypertens*. 2021;39:1361-1369

34. Regensteiner JG, Bauer TA, Huebschmann AG, Herlache L, Weinberger HD, Wolfel EE, et al. Sex differences in the effects of type 2 diabetes on exercise performance. *Med Sci Sports Exerc*. 2015;47:58-65

35. Province VM, Szeghy RE, Stute NL, Augenreich MA, Behrens CE, Stickford JL, et al. Tracking peripheral vascular function for six months in young adults following sars-cov-2 infection. *Physiol Rep*. 2022;10:e15552

36. Renata S, Tomasz D, Andrzej K, Sławomir T. Impact of 10 sessions of whole body cryostimulation on cutaneous microcirculation measured by laser doppler flowmetry. *J Hum Kinet*. 2011;30:75-83

37. Yvonne-Tee GB, Rasool AH, Halim AS, Wong AR, Rahman AR. Method optimization on the use of postocclusive hyperemia model to assess microvascular function. *Clin Hemorheol Microcirc*. 2008;38:119-133

38. Stupin A, Stupin M, Baric L, Matic A, Kolar L, Drenjancevic I. Sex-related differences in forearm skin microvascular reactivity of young healthy subjects. *Clin Hemorheol Microcirc*. 2019;72:339-351

39. Rodrigues LM, Pinto PC, Leal A. Transcutaneous flow related variables measured in vivo: The effects of gender. *BMC Dermatol*. 2001;1:4

40. Migdal KU, Robinson AT, Watso JC, Babcock MC, Lennon SL, Martens CR, et al. A high salt meal does not impair cerebrovascular reactivity in healthy young adults. *Physiol Rep*. 2020;8:e14585

41. Yang PT, Yuan H, Wang YQ, Cao X, Wu LX, Chen ZH. Correlations between brachial endothelial function and cardiovascular risk factors: A survey of 2,511 chinese subjects. *J Thorac Dis*. 2014;6:1441-1451

42. Celermajer DS, Sorensen KE, Spiegelhalter DJ, Georgakopoulos D, Robinson J, Deanfield JE. Aging is associated with endothelial dysfunction in healthy men years before the age-related decline in women. *J Am Coll Cardiol*. 1994;24:471-476

43. Perregaux D, Chaudhuri A, Rao S, Airen A, Wilson M, Sung BH, et al. Brachial vascular reactivity in blacks. *Hypertension*. 2000;36:866-871

44. Hamburg NM, Charbonneau F, Gerhard-Herman M, Ganz P, Creager MA. Comparison of endothelial function in young men and women with a family history of premature coronary artery disease. *Am J Cardiol*. 2004;94:783-785

45. Hurks R, Eisinger MJ, Goovaerts I, van Gaal L, Vrints C, Weyler J, et al. Early endothelial dysfunction in young type 1 diabetics. *Eur J Vasc Endovasc Surg*. 2009;37:611-615

46. Lin M, Lin SL, Wang KL, Kuo HW, Tak T. Effect of aging on human circulatory system in normotensive healthy subjects. *Int J Angiol*. 2014;23:233-242

47. Perregaux D, Chaudhuri A, Mohanty P, Bukhari L, Wilson MF, Sung BH, et al. Effect of gender differences and estrogen replacement therapy on vascular reactivity. *Metabolism*. 1999;48:227-232

48. Corretti MC, Plotnick GD, Vogel RA. The effects of age and gender on brachial artery endothelium-dependent vasoactivity are stimulus-dependent. *Clin Cardiol*. 1995;18:471-476

49. Lan WR, Hou CJ, Yen CH, Shih BF, Wang AM, Lee TY, et al. Effects of carbenoxolone on flow-mediated vasodilatation in healthy adults. *Am J Physiol Heart Circ Physiol*. 2011;301:H1166-1172

50. Paradossi U, Ciofini E, Clerico A, Botto N, Biagini A, Colombo MG. Endothelial function and carotid intima-media thickness in young healthy subjects among endothelial nitric oxide synthase glu298-->asp and t-786-->c polymorphisms. *Stroke*. 2004;35:1305-1309

51. Tremblay JC, Stimpson TV, Pyke KE. Evidence of sex differences in the acute impact of oscillatory shear stress on endothelial function. *J Appl Physiol (1985)*. 2019;126:314-321

52. Tremblay JC, Grewal AS, Pyke KE. Examining the acute effects of retrograde versus low mean shear rate on flow-mediated dilation. *J Appl Physiol (1985)*. 2019;126:1335-1342

53. Nair BM, Viswanathan V, Snehalatha C, Mohan RS, Ramachandran A. Flow mediated dilatation and carotid intimal media thickness in south indian type 2 diabetic subjects. *Diabetes Res Clin Pract*. 2004;65:13-19

54. Mashayekhi G, Zahedi E, Movahedian Attar H, Sharifi F. Flow mediated dilation with photoplethysmography as a substitute for ultrasonic imaging. *Physiol Meas*. 2015;36:1551-1571

55. Parker BA, Tschakovsky ME, Augeri AL, Polk DM, Thompson PD, Kiernan FJ. Heterogenous vasodilator pathways underlie flow-mediated dilation in men and women. *Am J Physiol Heart Circ Physiol*. 2011;301:H1118-1126

56. Hanson BE, Iwamoto E, Mouser BL, Miller KA, Casey DP. Hypoxia offsets the decline in brachial artery flow-mediated dilation after acute inactivity. *Am J Physiol Regul Integr Comp Physiol*. 2022;323:R787-r796

57. Hwang IC, Kim KH, Choi WS, Kim HJ, Im MS, Kim YJ, et al. Impact of acute exercise on brachial artery flow-mediated dilatation in young healthy people. *Cardiovasc Ultrasound*. 2012;10:39

58. Jiang XB, Li CL, He DS, Mao ZG, Liu DH, Fan X, et al. Increased carotid intima media thickness is associated with prolactin levels in subjects with untreated prolactinoma: A pilot study. *Pituitary*. 2014;17:232-239

59. Lindkvist M, Fernberg U, Ljungberg LU, Fälker K, Fernström M, Hurtig-Wennlöf A, et al. Individual variations in platelet reactivity towards adp, epinephrine, collagen and nitric oxide, and the association to arterial function in young, healthy adults. *Thromb Res*. 2019;174:5-12

60. Petrovic DJ. The influence of aging-related decrease in endothelial function on the stiffness of peripheral conducting arteries. *Journal for Vascular Ultrasound*. 2023;47:184-192

61. Hanson BE, Casey DP. Intermittent versus continuous handgrip exercise and peripheral endothelial function: Impact of shear rate fluctuations. *J Appl Physiol (1985)*. 2023;135:892-901

62. Safdar B, Ali A, D'Onofrio G, Katz SD. Microvascular dysfunction as opposed to conduit artery disease explains sex-specific chest pain in emergency department patients with low to moderate cardiac risk. *Clin Ther*. 2016;38:240-255.e241

63. Barton M, Turner AT, Newens KJ, Williams CM, Thompson AK. Minimum recovery time between reactive hyperemia stimulus in the repeated measurement of brachial flow-mediated dilatation. *Ultrasound Med Biol*. 2011;37:879-883

64. Hashimoto M, Akishita M, Eto M, Ishikawa M, Kozaki K, Toba K, et al. Modulation of endothelium-dependent flow-mediated dilatation of the brachial artery by sex and menstrual cycle. *Circulation*. 1995;92:3431-3435

65. Uehata A, Lieberman EH, Gerhard MD, Anderson TJ, Ganz P, Polak JF, et al. Noninvasive assessment of endothelium-dependent flow-mediated dilation of the brachial artery. *Vasc Med*. 1997;2:87-92

66. Celermajer DS, Adams MR, Clarkson P, Robinson J, McCredie R, Donald A, et al. Passive smoking and impaired endothelium-dependent arterial dilatation in healthy young adults. *N Engl J Med*. 1996;334:150-154

67. Campia U, Choucair WK, Bryant MB, Waclawiw MA, Cardillo C, Panza JA. Reduced endothelium-dependent and -independent dilation of conductance arteries in african americans. *J Am Coll Cardiol*. 2002;40:754-760

68. Nishiyama SK, Wray DW, Richardson RS. Sex and limb-specific ischemic reperfusion and vascular reactivity. *Am J Physiol Heart Circ Physiol*. 2008;295:H1100-h1108

69. Ratchford SM, Stickford JL, Province VM, Stute N, Augenreich MA, Koontz LK, et al. Vascular alterations among young adults with sars-cov-2. *Am J Physiol Heart Circ Physiol*. 2021;320:H404-h410

70. Paradossi U, Manfrini O, Ciofini E, Pizzi C, Biagini A, Xhyheri B, et al. Weight is an independent predictor of vascular injury in healthy volunteers with aspartate allele. *J Cardiovasc Med (Hagerstown)*. 2012;13:307-312

71. Akins JD, Martin ZT, Patik JC, Curtis BM, Campbell JC, Olvera G, et al. Young, non-hispanic black men and women exhibit divergent peripheral and cerebral vascular reactivity. *Exp Physiol*. 2022;107:450-461

72. Plotnick GD, Corretti MC, Vogel RA. Effect of antioxidant vitamins on the transient impairment of endothelium-dependent brachial artery vasoactivity following a single high-fat meal. *Jama*. 1997;278:1682-1686

73. Germain AM, Irribarra V, Valdés G, Romanik MC, Leighton F, Mardones F, et al. [ultrasound assessment of endothelial function in chilean children and adults]. *Rev Med Chil*. 2004;132:437-444

74. Shenouda N, Skelly LE, Gibala MJ, MacDonald MJ. Brachial artery endothelial function is unchanged after acute sprint interval exercise in sedentary men and women. *Exp Physiol*. 2018;103:968-975

75. Soares C, Koch H, Montenegro C, Rezende-Filho J, Leite S, Braga A, et al. Brachial artery flow-mediated dilatation and intima-media thickness of carotid and brachial arteries: Evaluation of individuals with and without risk factors for atherosclerosis. *Radiol. Bras.* 2010;43:389-393

76. Nakamura A, Kajitani S, Sato K, Kanazawa M, Kondo M, Endo H, et al. Decline of popliteal artery flow-mediated dilation with aging and possible involvement of asymmetric dimethylarginine in healthy men. *J Med Ultrason (2001)*. 2019;46:503-511

77. Black MA, Cable NT, Thijssen DH, Green DJ. Impact of age, sex, and exercise on brachial artery flow-mediated dilatation. *Am J Physiol Heart Circ Physiol*. 2009;297:H1109-1116

78. Skow RJ, Nandadeva D, Grotle AK, Stephens BY, Wright AN, Fadel PJ. Impact of breakthrough covid-19 cases during the omicron wave on vascular health and cardiac autonomic function in young adults. *Am J Physiol Heart Circ Physiol*. 2022;323:H59-h64

79. Morishima T, Padilla J, Tsuchiya Y, Ochi E. Maintenance of endothelial function following acute resistance exercise in females is associated with a tempered blood pressure response. *J Appl Physiol (1985)*. 2020;129:792-799

80. Harris RA, Tedjasaputra V, Zhao J, Richardson RS. Premenopausal women exhibit an inherent protection of endothelial function following a high-fat meal. *Reprod Sci*. 2012;19:221-228

81. Holder SM, Brislane Á, Dawson EA, Hopkins ND, Hopman MTE, Cable NT, et al. Relationship between endothelial function and the eliciting shear stress stimulus in women: Changes across the lifespan differ to men. *J Am Heart Assoc*. 2019;8:e010994

82. Lennon-Edwards S, Ramick MG, Matthews EL, Brian MS, Farquhar WB, Edwards DG. Salt loading has a more deleterious effect on flow-mediated dilation in salt-resistant men than women. *Nutr Metab Cardiovasc Dis*. 2014;24:990-995

83. Gentilin A, Moghetti P, Cevese A, Schena F, Tarperi C. Sympathetic-mediated blunting of forearm vasodilation is similar between young men and women. *Biol Sex Differ*. 2022;13:33

84. Badrov MB, Freeman SR, Zokvic MA, Millar PJ, McGowan CL. Isometric exercise training lowers resting blood pressure and improves local brachial artery flow-mediated dilation equally in men and women. *Eur J Appl Physiol*. 2016;116:1289-1296

85. Arbeitman CR, Cymberknop LJ, Farro I, Cardelino J, Armentano RL. Vascular reactivity in healthy subjects: Simultaneous characterization of arterial pressure and diameter time profiles. *Health Technol*. 2016;6:189-195

86. King TJ, Petrick HL, Millar PJ, Burr JF. Acute oral antioxidant consumption does not alter brachial artery flow mediated dilation in young adults independent of exercise training status. *Appl Physiol Nutr Metab*. 2024;49:375-384

87. Tomiyama H, Matsumoto C, Yamada J, Teramoto T, Abe K, Ohta H, et al. The relationships of cardiovascular disease risk factors to flow-mediated dilatation in japanese subjects free of cardiovascular disease. *Hypertens Res*. 2008;31:2019-2025

88. Kim WS, Hwang SJ, Na HY, Kang HS, Jo JH, Kim GS, et al. Evaluation of endotheial function in normal korean adults and in patients with essential hypertension. *jkse*. 2000;8:59-70

89. Smiljanec K, Mbakwe AU, Ramos-Gonzalez M, Pohlig RT, Lennon SL. Antioxidant cocktail following a high-sodium meal does not affect vascular function in young, healthy adult humans: A randomized controlled crossover trial. *Nutr Res*. 2020;79:13-22

90. Turner CG, Walker DC, Wong BJ. Contribution of sensory nerves to cutaneous reactive hyperaemia in non-hispanic black and white young adults. *Exp Physiol*. 2023;108:802-809

91. Fujii N, McGarr GW, Amano T, Boulay P, Nishiyasu T, Kenny GP. Does aging alter skin vascular function in humans when spatial variation is considered? *Microcirculation*. 2022;29:e12743

92. Silva H, Ferreira H, Bujan MJ, Rodrigues LM. Regarding the quantification of peripheral microcirculation--comparing responses evoked in the in vivo human lower limb by postural changes, suprasystolic occlusion and oxygen breathing. *Microvasc Res*. 2015;99:110-117
